# A protective, single-visit TB vaccination regimen by co-administration of a subunit vaccine with BCG

**DOI:** 10.1038/s41541-023-00666-2

**Published:** 2023-05-09

**Authors:** Karin Dijkman, Thomas Lindenstrøm, Ida Rosenkrands, Rikke Søe, Joshua S. Woodworth, Cecilia S. Lindestam Arlehamn, Rasmus Mortensen

**Affiliations:** 1 Department of Infectious Disease Immunology, Statens Serum Institut, Copenhagen, Denmark; 2 Department of Vaccine Development, Statens Serum Institut, Copenhagen, Denmark; 3 Center for Infectious Disease and Vaccine Research, La Jolla Institute for Immunology, La Jolla, CA USA; 4 Present Address: Janssen Vaccines & Prevention, Leiden, the Netherlands

**Keywords:** Tuberculosis, Protein vaccines, Tuberculosis

## Abstract

The only licensed tuberculosis (TB) vaccine, Bacillus Calmette Guerin (BCG), fails to reliably protect adolescents and adults from pulmonary TB, resulting in ~1.6 million deaths annually. Protein subunit vaccines have shown promise against TB in clinical studies. Unfortunately, most subunit vaccines require multiple administrations, which increases the risk of loss to follow-up and necessitates more complex and costly logistics. Given the well-documented adjuvant effect of BCG, we hypothesized that BCG co-administration could compensate for a reduced number of subunit vaccinations. To explore this, we developed an expression-optimized version of our H107 vaccine candidate (H107e), which does not cross-react with BCG. In the CAF®01 adjuvant, a single dose of H107e induced inferior protection compared to three H107e/CAF®01 administrations. However, co-administering a single dose of H107e/CAF®01 with BCG significantly improved protection, which was equal to BCG co-administered with three H107e/CAF®01 doses. Importantly, combining BCG with a single H107e/CAF®01 dose also increased protection in previously BCG-primed animals. Overall, a single dose of H107e/CAF®01 with BCG induced long-lived immunity and triggered BCG-specific Th17 responses. These data support co-administration of BCG and subunit vaccines in both BCG naïve and BCG-primed individuals as an improved TB vaccine strategy with reduced number of vaccination visits.

## Introduction

The worldwide disruption of healthcare systems due to the Covid-19 pandemic has reversed years of progress in the fight against tuberculosis (TB). After a steady decline since 2005, the number of TB deaths increased in both 2020 and 2021 to reach 1.6 million and TB mortality is expected to rise further in the coming years due to reduced access to diagnosis and treatment^1^. While there is a licensed TB vaccine available, the live attenuated Bacillus Calmette Guerin (BCG), it fails to reliably protect adults from pulmonary TB, which is the main driver of TB transmission^2^. As such, more efficient vaccination strategies are urgently required. Two clinical trials on adjuvanted protein subunit vaccines have reported encouraging efficacy results^3,4^ which offers the perspective of an improved TB vaccine regimen utilizing such vaccines.

However, a subunit vaccination regimen typically requires multiple booster vaccinations to confer maximum efficacy, which is suboptimal in TB-endemic countries. Compared to the single-visit BCG regimen, a multi-visit regimen is vulnerable to loss of follow-up, requires more complex logistics and will be costlier to execute. As such, the WHO advises a vaccination schedule consisting of a minimal number of administrations as a Preferred Product Characteristic for novel TB vaccines^5^. Therefore, an ideal TB vaccination regimen would consist of a single administration for maximum coverage at minimal cost. Several studies support the notion that a single-dose vaccination regimen has the potential to be durably efficacious against other diseases^6–11^.

Single-visit Mtb vaccination strategies can be achieved by the use of live attenuated Mtb vaccines or novel recombinant BCG strains engineered to broaden and/or increase immune responses through the insertion of genes for Mtb-specific antigens or host factors. However, in such settings, all antigens would be expected to receive the same “imprint” leading to immune cells of the same functionality. In addition, molecular insertion of Mtb-specific antigens inherently carries the risk of rendering the recombinant strains more virulent. Another approach to enable a single-visit vaccination regimen against TB could be to leverage the existing BCG vaccine regimen through co-administration of BCG with a subunit vaccine to simultaneously boost and broaden immunogenicity. Beyond conferring protection against pediatric TB disease, BCG has several immunomodulatory properties. The most well-known is its ability to confer heterologous protection through mechanisms such as trained immunity^12,13^. However, when co-administered with other vaccines, either simultaneously or closely together in time, it has also been reported to improve heterologous immune responses by a “co-adjuvanting” effect^13–16^. Recently it was shown that co-administration of a single dose of BCG with trimeric SARS-Cov-2 spike, combined with alum, provided K18-hACE2 mice with sterilizing immunity against experimental SARS-Cov-2 challenge^17^. This ability of BCG to potentiate vaccine efficacy prompted us to investigate its potential to adjuvant TB vaccine responses.

There are currently a number of TB subunit vaccines in various stages of clinical development and virtually all of them share some or all of their antigens with BCG. This makes them less suited as candidates for a single-dose co-administration regimen with BCG, as the BCG-reactive immune responses elicited by the subunit vaccine could interfere with BCG persistence and immunogenicity. However, as part of our TB vaccine development program, we have designed the H107 fusion protein that consists of 8 antigens specifically selected for not being expressed and/or secreted by BCG and therefore not to interfere with BCG-mediated immune responses^18^. H107 is therefore ideal for co-administration with BCG. Importantly, in a three-dose regimen, co-administration of BCG with the first dose of H107 leads to increased T-cell responses against both H107 and BCG^18^. We hypothesized that this mutual adjuvant effect could compensate for a reduced number of vaccine administrations and open up the possibility of an efficacious single-visit regimen.

Here we introduce a variant of the H107 fusion antigen, H107e, optimized for increased protein expression. We investigate the immunogenicity and efficacy of BCG co-administered with a single dose of H107e in the CAF®01 adjuvant^19^ (BCG+1xH107e) and show that this induces long-lived T cell responses, albeit at lower magnitude than BCG+3xH107e. Regardless, in a mouse model of tuberculosis, BCG+1xH107e increased long-term protection over both 1x and 3xH107e/CAF®01 and was as protective as BCG+3xH107e co-administration. Importantly, in a BCG revaccination model, co-administering BCG with a single dose of H107e also significantly enhanced long-term protection. Dissection of the immune responses of BCG+1xH107e prior to the challenge revealed an altered innate cellular composition in the vaccine-draining lymph node compared to BCG immunization alone, which was associated with a Th17-skewing of the BCG-specific T cell response. This Th17-biased BCG response was markedly expanded post-challenge. Recognizing the increasing body of literature supporting a protective role for Th17 cells^20–25^, these results warrant further investigation. Ultimately, an efficacious single-dose TB vaccine regimen has the potential to lower implementation complexity, reduce costs and increase global vaccine coverage.

## Results

### A modified construct, H107e, displays enhanced protein expression and synergizes with BCG co-administration

**Fig. 1:**
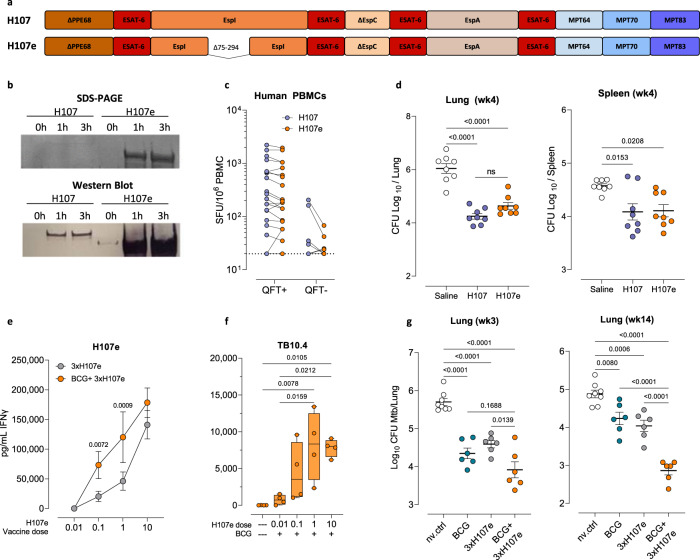
H107e displays enhanced protein expression and synergizes with BCG co-administration. **a** Antigen design of the H107e construct. H107e was modified from H107 through deletion of a proline-rich fraction of 220 AA in the Espl/Rv3876 antigen (Δ75–294). **b** Expression of the H107 and H107e constructs (OD adjusted cultures) as shown by SDS-PAGE (upper) and Western Blot (lower) at 0, 1-, and 3-h post-induction. Protein purity was estimated to be above 95% based on SDS-page followed by Coomassie staining and an anti-E. coli western blot. H107e had a recovery yield of 2.5 – 4.0 mg/L culture media, which was 2.5–5-fold higher than for H107. **c** Magnitude of IFN-γ T cell response (SFC/10^6^ PBMCs) in healthy QFT+ (*n* = 22) and QFT- (*n* = 10) subjects against H107 (blue dots) and H107e (orange dots). Dotted line indicates the limit of detection at 20 SFC/10^6^ PBMCs. Two-tailed unpaired t-test. **d** Bacterial burden in lung (left) and spleen (right) determined four weeks after aerosol challenge with Mtb Erdman in mice immunized with either 3xH107/CAF®01 or 3xH107e/CAF®01. Data plotted as mean ± SEM (*n* = 8). One-Way ANOVA with Tukey’s multiple comparisons test. One of two representative experiments shown. H107e-specific (**e**) and TB10.4-specific (**f**) IFN-γ response of splenocytes 6 weeks post immunizations with H107e doses ranging from 0.01 µg to 10 µg. Levels of IFN-γ shown as mean ± SEM (**e**) and as box plots showing median ± IQR with whiskers signifying the range of responses (**f**). Symbols indicate individual mice (*n* = 4). One-Way ANOVA with Fisher’s LSD for comparing H107e responses with or without BCG co-administration for each given H107e dose (**e**) and Tukey’s multiple comparison test for TB10.4 responses (**f**). **g** Lung bacterial burden at week three (left) and fourteen (right) post aerosol challenge with Mtb Erdman in mice H107e/CAF®01 immunized with or without BCG co-administration. Data plotted as mean ± SEM (*n* = 6–8); One-Way ANOVA with Tukey’s multiple comparisons test.

H107 consists of eight selected antigens with confirmed human immune recognition and proven protection in animal models^18^. However, the expression of H107 in *E. coli* is low, hampering further clinical development. EspI/Rv3876 is one of the antigens in H107, and challenges with purifying full-length espI/Rv3876 have previously been described and may be explained by a high content of disorder segments in the first 400 amino acids with abundant alanine and proline residues^26^. In order to enable large-scale expression, we generated a modified construct, H107e, by selectively deleting a proline-rich fraction in the Espl/Rv3876 antigen (Δ75-294; Fig. 1a). This modification led to highly increased expression of H107e compared to H107 as shown by SDS-PAGE and Western Blotting (Fig. 1b, full SDS-page gel in Supplementary Fig. 1b). Protein purity of H107e was estimated to be above 95% based on SDS-page followed by Coomassie staining and an anti-E. coli western blot. H107e had a recovery yield of 2.5–4.0 mg/L culture media, which translated to a 2.5–5-fold higher yield recovery than for H107 measured by the bicinchoninic acid protein assay. Of importance, the deleted fraction (Δ75–294) only contained 4 out of 40 predicted HLA-binding epitopes within the Espl/Rv3876 antigen (Supplementary Fig. 1a), and the deletion in H107e did not affect immunological recognition in healthy Quantiferon (QFT) positive human subjects (Fig. 1c). Likewise, H107e was as immunogenic as H107 in mice and elicited similar levels of IFN-γ recall responses (Supplementary Fig. 1c), though with slightly lower recall responses against full-length Espl/Rv3876 and a trend towards increased responses against MPT70 and MPT83 (Supplementary Fig. 1d). In accordance, 3 administrations of H107e induced similar protection as H107, 4 weeks after experimental challenge with *Mycobacterium tuberculosis* (Mtb) Erdman (Fig. 1d).

In our previous report, we showed that BCG + H107 co-administration enhances the immunogenicity of both vaccines and improves long-term protection^18^. Therefore, we investigated whether this was also the case for H107e. Indeed, co-administering BCG with three doses of H107e in CAF®01 (BCG+3xH107e) significantly increased the H107e-specific T cell response over three times H107e in CAF®01 (Supplementary Fig. 1e), which was evident over a range of H107e doses (Fig. 1e). Similarly, H107e/CAF®01 co-administration also increased BCG-specific responses (measured by IFNγ-release after stimulation with the dominant TB10.4/Rv0288 antigen in BCG) in dose ranges of 0.1–10 µg H107e, indicating true reciprocal adjuvanticity (Fig. 1f). Finally, we confirmed that BCG+3xH107e significantly improved protection over 3xH107e at both 3 and 14 weeks post challenge (Fig. 1g). This increase in protection could be observed in a dose range of 0.01–10 µg H107e at week 3, whereas co-administration of 0.01–1 µg H107e improved protective efficacy at late stage infection (week 14) (Supplementary Fig. 1f).

Collectively, these results show that the modifications in H107e led to significantly enhanced protein expression while maintaining immunogenicity and protective capacity. Most importantly, H107e+BCG co-administration significantly reduced Mtb loads, with lower doses being optimal for long-term protection.

### BCG+1xH107e co-administration provides long-term protection on par with BCG+3xH107e

**Fig. 2:**
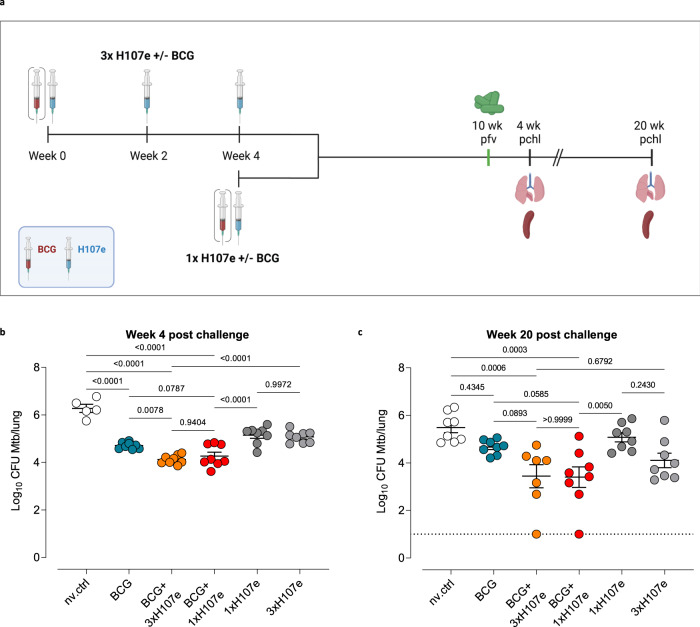
BCG+1xH107e co-administration provides long-term protection on par with BCG+3xH107e. **a** Study design comparing short- and long-term protective efficacy of a 3-dose versus 1-dose BCG+H107e/CAF®01 co-administration regimen. Red syringe: BCG; blue syringe: H107e/CAF®01. pfv: post final vaccination, pchl: post challenge. Created with Biorender. **b** Lung bacterial loads 4 weeks after Mtb challenge. **c** Lung bacterial loads 20 weeks after Mtb challenge. In (**b**) and (**c**), line and error bars indicate mean and SEM (*n* = 5–8). Statistical significance of group differences was determined via one-way ANOVA and Tukey’s multiple comparison test.

The heterologous design of H107e and its ability to complement BCG prompted us to investigate if we could leverage this synergy towards a single dose TB vaccine regimen. To address this, mice were either immunized 3 times with H107e/CAF®01, with or without BCG co administered with the first vaccination (±BCG+3xH107e) or 1 time with H107e/CAF®01 with or without BCG (±BCG+1xH107e) and exposed to Mtb by the aerosol route 10 weeks after the last immunization (Fig. 2a). A BCG only group was included as a benchmark for protective efficacy and bacterial burdens were determined at 4 and 20 weeks post infection (p.i.). At week 4 p.i., all vaccination regimens significantly lowered the bacterial lung burdens with a −Δlog10 in the range of 1 – 2.1 relative to non-vaccinated controls (Fig. 2b). As expected, BCG co-administration significantly improved protection compared to both 1x and 3xH107e alone as well as BCG alone. However, to our surprise, the co-administration schedules with single and triple administrations (BCG+1xH107e vs BCG+3xH107e) resulted in similar levels of protection (Fig. 2b). Same findings were seen in an independent repeat study (Supplementary Fig. 2). Furthermore, at late-stage infection, 20 weeks p.i., where the protective efficacy of BCG and 1xH107e alone waned, the BCG+1xH107e and the BCG+3xH107e protected equally well and both induced >2 log10 reductions in bacterial load compared to non-vaccinated controls (Fig. 2c). A similar pattern could be observed in the spleen, where BCG+1xH107e provided equal protection to the BCG+3xH107e vaccine schedule and elicited significant protection over BCG vaccinated mice at week 20 p.i. (Supplementary Fig. 3).

Overall, these data demonstrated that BCG+1xH107e co-administration provides long-term protection on par with the BCG+3xH107e regimen (Fig. 2c).

### BCG+1xH107e provides long-term protection in previously BCG-vaccinated animals

**Fig. 3:**
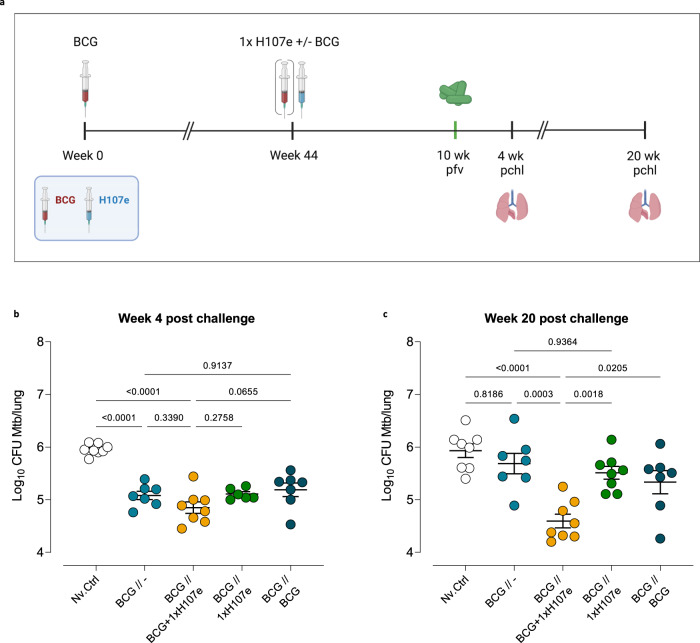
BCG+1xH107e provides increased long-term protection in previously BCG-vaccinated animals. **a** Study design comparing short- and long-term protective efficacy of a 1-dose BCG+H107e/CAF®01 co-administration regimen in mice previously vaccinated with BCG. Red syringe BCG, blue syringe H107e/CAF01. pfv post final vaccination, pchl post challenge. Created with Biorender. **b** Lung bacterial loads 4 weeks after Mtb challenge. **c** Lung bacterial loads 20 weeks after Mtb challenge. In (**b**) and (**c**), line and error bars indicate mean and SEM (*n* = 6–8). Statistical significance of group differences was determined via one-way ANOVA and Tukey’s multiple comparison test.

Recent clinical data showing a potential protective effect of revaccination with BCG has generated renewed interest in BCG revaccination^3^. As preexisting immunity to BCG could potentially decrease the protective efficacy of our co-administration regimen, we next asked to what extent BCG+1xH107e protected in a revaccination setting. In order to mimic a setting of distal BCG, BCG-vaccinated mice were rested for 44 weeks before immunization with 1xH107e ± BCG. A BCG revaccination group was included for reference. Ten weeks after immunization, mice were challenged with Mtb Erdman via aerosol and protective efficacy was measured 4 and 20 weeks after infection (Fig. 3a). At week 4 post-challenge, distal BCG vaccination still provided significant protection over non-vaccinated controls. However, BCG revaccination did not add to the protection afforded by distal BCG. Likewise, 1xH107e, either with or without BCG co-administration, did not lead to further reductions in lung bacterial burden at the early time point (Fig. 3b). However, at late-stage infection, 20 weeks post-challenge, the protective effect of distal BCG had waned and did not significantly reduce lung bacterial load relative to non-vaccinated mice. Revaccination with BCG or vaccination with 1xH107e could not counteract this decline. However, BCG+1xH107e remained significantly protective at week 20 compared to both saline controls and mice previously vaccinated with BCG (Fig. 3c). A similar pattern could be discerned in the spleen (Supplementary Fig. 3).

In summary, this data demonstrated that pre-existing immunity to BCG does not compromise the synergy in the H107e+BCG co-administration regimen and that BCG+1xH107e induces a long-lasting protective response in a BCG revaccination setting.

### BCG+1xH107e induces long-lived H107e-specific CD4 T cells responses

**Fig. 4:**
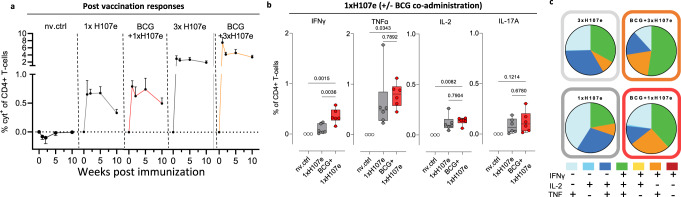
BCG+1xH107e induces long-lived H107e-specific CD4 T cell responses. **a** Cumulative frequency of cytokine-positive (IFN-γ, TNF, IL-2, and/or IL-17A) splenocytes after stimulation with H107e protein at various weeks post final vaccination. Frequencies plotted as group mean + SEM (*n* = 6, though *n* = 3 in nv. ctrl (non-vaccinated control)). **b** Comparison of individual cytokine responses between the animals vaccinated with 1xH107e versus BCG+1xH107e, 1 week after vaccination. Data graphed as box plots with line at median, boxes indicating interquartile range, and whiskers delineating minimum and maximum values. Statistical significance of group differences determined via unpaired t-test. *n* = 6, though *n* = 3 in nv. ctrl (non-vaccinated control). **c** Boolean gating analysis of IFN-γ, TNF, and IL-2 positivity of antigen-specific CD4 T cells for each immunized group, 1 week post-final vaccination. Pies indicate average proportion of each combination of cytokine expression; *n* = 6.

To explore what could underlie the enhanced protective effect conferred by BCG and H107e co-administration, we next turned to characterize the H107e specific responses in terms of immunogenicity and phenotype as induced by a 1x vs 3xH107e regimen either with or without BCG co-administration. H107e-specific immune responses were assessed at various weeks after vaccination by antigen stimulation of splenocytes followed by intracellular cytokine staining (ICS) for IFN-γ, TNF, IL-2, and IL-17A. We found 1xH107e to be immunogenic, with long-lasting CD4 T cell responses both with and without BCG co-administration (Fig. 4a). The adjuvant effect of BCG-co-administration on H107e-specific immune responses was less apparent compared to the three-dose regimen, but with a tendency for lower contraction from week 5 to 10 in the BCG+1xH107e group compared to 1xH107e (Fig. 4a). Focusing on individual cytokines, the vaccine regimens primed H107e-specific CD4 T cells expressing all of the four cytokines, including IL-17. Comparing the response magnitudes between 1xH107e and BCG+1xH107e revealed that the only difference between the two groups was in the frequency of IFN-γ-producing CD4 T cells, whereas the levels of TNF, IL-2, and IL-17A-producing subsets were similar (Fig. 4b, Supplementary Fig. 4a). In the three-dose regimen, the immune responses were substantially higher than in the single dose regimen and here, the co-adjuvant effect of BCG was more evident, in line with previous studies^15,18^ (Fig. 4a and Supplementary Fig. 4b). Combinatorial expression analysis of the vaccine-primed Th1 cytokines showed no significant differences between the 1x vs. 3x regimens (Fig. 4c), highlighting that the responses after BCG+1xH107e vs. BCG+3xH107e immunization only differed in magnitude and not longevity/phenotype. Analysis of the H107e-specific lung CD4 T cells at week 4 post Mtb challenge showed significant expansion compared to pre-challenge, but confirmed that responses between BCG+1xH107e and 1xH107e also were similar after Mtb infection (Supplementary Fig. 4c).

In summary, BCG+1xH107e induced robust and long-lived H107e-specific CD4 T cell responses with a similar phenotype as BCG+3xH107e, albeit at a lower magnitude. Given the similar protection levels between BCG+1xH107e and BCG+3xH107e, these findings suggest that the magnitude of the H107e immune response is not critical for the protection in the co-administration regimen.

### BCG+1xH107e co-administration skews BCG-specific immune responses towards a Th17 phenotype

**Fig. 5:**
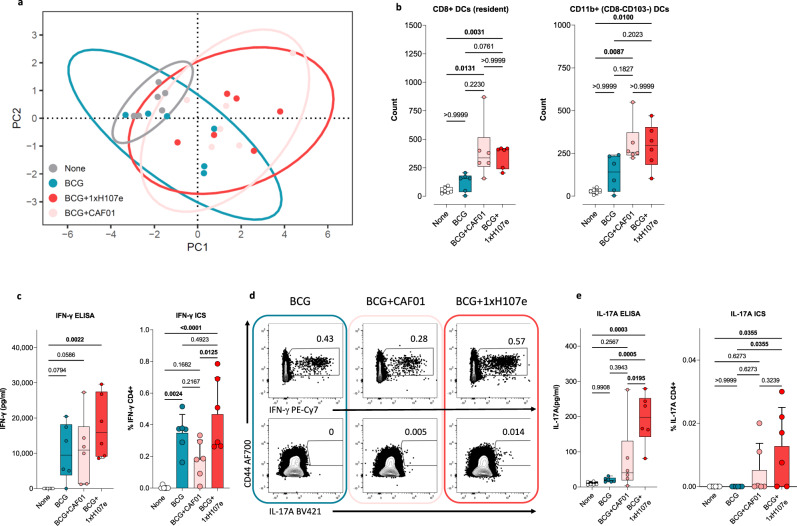
BCG+1xH107e co-administration induces BCG-specific Th17 cells. **a–e** Pre-challenge responses at 5 weeks post vaccination. **a** Principal component analysis (PCA) of neutrophil, Ly6C- and Ly6C+ monocyte, CD8a+-resident, CD103+-migratory, CD11b+, and Ly6C+ DC cell numbers in vaccine-draining lymph nodes among BCG (blue), BCG + CAF01 (pink) and BCG+1x H107e (red) immunized relative to non-immunized control mice (None; gray). **b** Number of CD8a+ resident (left) and CD11b+ (right) DCs. Box plots showing median ± IQR with whiskers signifying the range of responses. Statistically significant differences between groups determined using Kruskall–Wallis and Dunn’s multiple comparison test, *n* = 6. **c** Magnitude of TB10.4-specific IFN-γ response from splenocytes. Box plots (left) show median ± IQR with whiskers covering range of TB10.4-specific IFN-γ-release as measured by ELISA. Kruskall-Wallis and Dunn’s multiple comparison test. Right bar chart shows the frequency of TB10.4-specific IFN-γ + CD4 T cells, One-way ANOVA with Tukey’s multiple comparison test. n = 6 in both cases. **d** Concatenated FACS plots of CD4 T cells showing the frequency of TB10.4-specific IFN-γ + (upper) and IL-17A+ (lower) cells among BCG (blue), BCG + CAF01 (pink) and BCG+1xH107e (red) immunized mice. **e** Magnitude of TB10.4-specific IL-17A release from splenocytes (left). Data shown as box plots with median ± IQR and whiskers covering range of responses. Right: frequency of TB10.4-specific IL-17A + CD4 T cells. Box plots showing median ± IQR with whiskers covering range of responses. In both cases, One-way ANOVA with Tukey’s multiple comparison test, *n* = 6.

Having established that H107e immunization can act as a reciprocal adjuvant for BCG-specific responses (Fig. 1f), we next asked to what extent BCG+1xH107e modified the cellular composition within the lymph node draining the injection site. To address this, we first conducted a small time-course study to identify the influx of neutrophils, inflammatory monocytes (Ly6C+Mo) and myeloid dendritic cells (CD11c) after BCG immunization (Supplementary Fig. 5a). Based on this pilot study, we found that peak influx of these innate cells happened around week 5, and we, therefore, phenotyped a wider range of innate immune cell populations including neutrophils, monocytes (Ly6C+ vs Ly6C-), and different dendritic cell (DC) subsets (CD8α+, as well as CD11b+ lymphoid DCs, migratory CD103+ DCs, and inflammatory Ly6C+ DCs) in the vaccine-draining inguinal lymph nodes at this time point. Principal component analysis (PCA) of the cell numbers of these populations revealed segregation among vaccinated (BCG, BCG + CAF01, and BCG+1xH107e) and unvaccinated mice, as well as clustering between BCG only and BCG+1xH107e animals, indicating an effect of H107e co-administration (Fig. 5a). The PCA loadings showed that a number of different DC subsets contributed to the discrimination between BCG on one hand and BCG + CAF01 and BCG+1xH107e on the other (Supplementary Fig. 5a). In particular, resident CD8a+ DCs, as well as CD11b+ DCs, appeared to be elevated in both BCG + CAF01 and BCG+1xH107e compared to BCG, although not reaching statistical significance (Fig. 5b). When examining the other different populations individually, inflammatory monocytes (Ly6C+Mo) were significantly induced after administration of all BCG containing vaccines, with no differences observed between the administration regimens (Supplementary Fig. 5d left). However, although neutrophils and Ly6C- monocytes were upregulated under both regimens, this was only significant after BCG+1xH107e immunization (Supplementary Fig. 5c and d right, respectively).

**Fig. 6:**
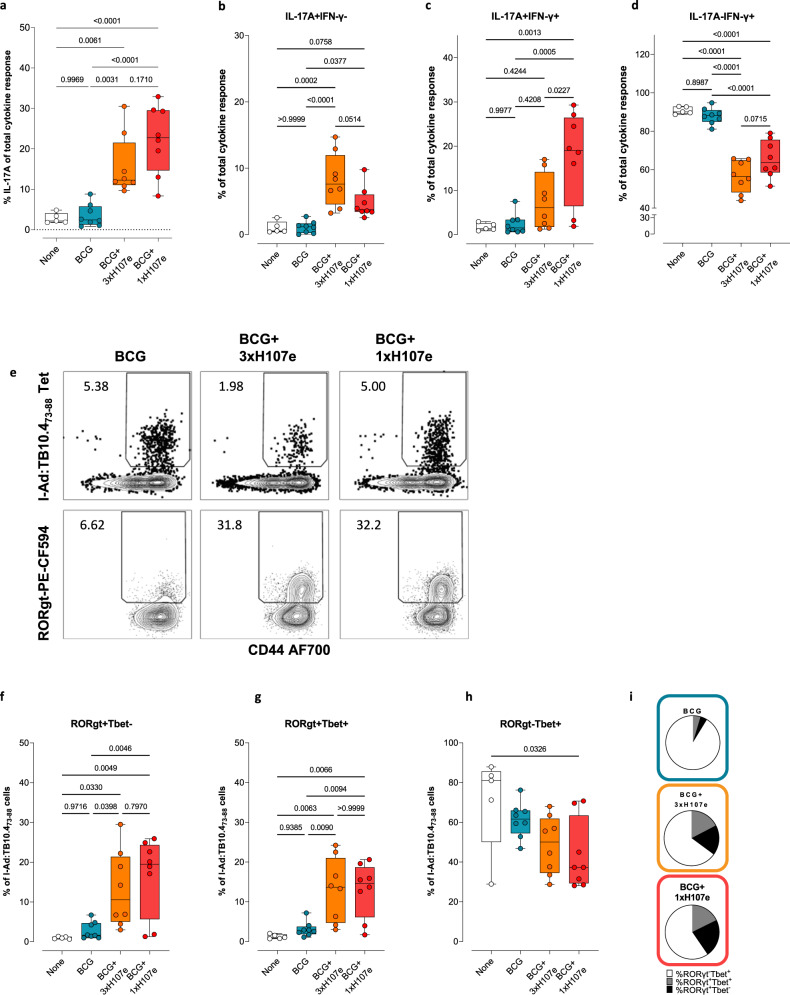
BCG-specific Th17 cells induced by BCG+H107e coadministration expand following challenge. Post-challenge lung responses in mice at week 4 post Mtb challenge. **a** Proportion of antigen-specific CD4 T cells (defined by expression of IFN-γ, TNF, IL-2, and/or IL-17A) expressing IL-17 after TB10.4 restimulation from mice analyzed at week 4 post Mtb infection. Box plots with median ± IQR and whiskers showing range of responses. One-way ANOVA with Tukey’s multiple comparison test, *n* = 5–8. Proportion of antigen-specific CD4 T cells (defined by expression of IFN-γ, TNF, IL-2, and/or IL-17A) being IL-17 + IFN-γ- (**b**), IL-17 + IFN-γ+ (**c**) IL-17-IFN-γ + (**d**) after TB10.4 restimulation from mice analyzed at week 4 post Mtb infection. Box plots with median ± IQR and whiskers showing range of responses. One-way ANOVA with Tukey’s multiple comparison test, n = 5–8. **e** Representative plots from indicated vaccine groups at week 4 post Mtb showing identification of antigen-specific lung CD44^hi^ CD4 T cells by virtue of I-A^d^:TB10.4_73–88_ MHC II tetramers (upper panels). Lower panels show frequencies of RORγt expressing cells among the TB10.4 Tetramer-positive T cells. Box plots showing the frequency of I-A^d^:TB10.4_73–88_ specific cells being RORγt^+^Tbet^-^ (**f**), RORγt^+^Tbet^+^ (**g**), and RORγt^-^Tbet^+^ (**h**). Data are shown as medians ± IQR with whiskers depicting range of responses. One-way ANOVA with Tukey’s multiple comparison test, *n* = 5–8. **i** Pie charts show the distribution of transcription factor expression among TB10.4 specific cells and illustrate the proportion of I-A^d^:TB10.4_73–88_ cells being either RORγt^-^Tbet^+^ (white pies), RORγt^+^Tbet^+^ (gray pies) or RORγt^+^Tbet^-^.

DCs are key players in T cell priming and differentiation. As the addition of CAF01 to BCG (with or without H107e) led to elevated numbers of CD8+ and CD11b+ DCs, we subsequently examined whether BCG + CAF01 and/or BCG+1xH107e affected the induction of BCG-specific CD4 and CD8 T cells by analyzing the magnitude and polarization of the TB10.4 responses. Here we observed a trend that BCG+1xH107e co-administration, but not BCG + CAF01, elevated TB10.4-specific IFN-γ responses as measured by IFN-γ release as well as flow cytometry for CD4 T cells (Fig. 5c, d). CD8 T cell induction was limited, and we found no signs of a CD8 adjuvanting effect in any of the groups (Supplementary Fig. 5f). More intriguingly, BCG+1xH107e co-administration was observed to skew the BCG-specific response towards a Th17 phenotype. Thus, from a complete absence of TB10.4-specific Th17 cells after BCG vaccination, 1xH107e co-administration primed significantly higher frequencies of IL-17A + CD4 T cells, measured by both flow cytometry (Fig. 5d, e) and IL-17A release (mean IL-17A: 193.8 ± 29.16 pg/ml relative to none, Fig. 5e). As the bias towards Th17 was not seen after immunization with BCG + CAF01, this polarization could not solely be ascribed to the addition of CAF01 (Fig. 5d, e). Thus, having established that the Th17 polarization of the BCG-specific response required supplementation of BCG with both adjuvant and antigen (CAF01 and H107e), we next turned to study if the single dose BCG+1xH107e could drive similar post-challenge responses to the BCG-specific TB10.4 antigen as the three-dose vaccination regimen (BCG+3xH107e). Notably, we found that the relatively modest magnitude of the Th17 response prior to Mtb infection was remarkably expanded post-challenge with no difference between the two co-administration schedules. Hence, the IL-17A proportion of the cytokine response after TB10.4 restimulation was significantly higher among mice that had received BCG+1xH107e or BCG+3xH107e compared to BCG only or non-vaccinated control mice (Fig. 6a). Looking at IL-17A/IFN-γ co-expression profiles, both co-administration groups exhibited significantly higher proportions of the TB10.4 specific cytokine response being IL-17A + IFN-γ- compared to BCG or non-vaccinated mice (Fig. 6b). In contrast, only the BCG+1xH107e groups had significantly elevated proportions of IL-17A + IFN-γ + relative to all other groups (Fig. 6c). In accordance with the Th17 skewing seen in both co-administration groups, the proportion of IL-17A-IFN-γ + Th1 cells were significantly lower than observed in the BCG and non-vaccinated mice (Fig. 6d). These findings were also reflected in the fraction of TB10.4 Tetramer-positive CD4 T cells expressing the Th17-related transcription factor RORγt (Fig. 6e), where ~6% of the TB10.4 Tet+ cells in BCG vaccinated mice expressed RORγt compared to nearly one third of the cells expressed this transcription factor after BCG+1xH107e or BCG+3xH107e, with no differences observed between these two groups (Fig. 6e). In terms of transcription factor co-expression, a significantly higher proportion of the TB10.4 Tet+ cells among the BCG+H107e co-administration groups were either RORgt+Tbet- (Th17) and ROTgt+Tbet+ (Th1/Th17), whereas Th1 cells (RORg-Tbet+) accordingly constituted a lower fraction Fig. 6f–i).

Taken together, these data demonstrated that co-administration of BCG with CAF01 (with or without H107e) altered the composition of innate immune populations in the draining lymph node. Analyzing the BCG-specific immune response, BCG+1xH107e co-administration lead to a switch from a strict Th1 response to a mixed Th1/Th17 response, which required the presence of both adjuvant and antigen. Importantly, BCG+1xH107e and BCG+3xH107e were found to be similar at driving this Th17 polarization following Mtb challenge.

## Discussion

In the slipstream of first-generation TB vaccines entering into advanced clinical stages, there is an increasing awareness about the current paucity of early-stage candidates in development. In order to bring improved TB vaccines to the forefront of the battle against TB, there is a recognized need to fill and diversify the TB vaccine pipeline. The H107 TB vaccine developed by Statens Serum Institut is a fusion protein composed of eight Mtb-specific antigens that do not cross-react with modern lineage BCG strains and is thus constructed to ‘complement’ the BCG response – either as a standalone vaccine or when co-administered with BCG^18^. However, due to its size and complexity, the expression of H107 in *E. coli* is challenging. In order to secure accessibility and cost-effectiveness, a high-yield manufacturing process of H107 is needed to enable large-scale expression. Here we introduce a high-expression version of H107, called H107e, which solves key issues with the expression of H107 by deleting a proline-rich stretch in the EspI antigen. Importantly, this deleted region contains few T cell epitopes predicted to bind HLA alleles and H107e was found to preserve both immunogenicity in mice and antigen recognition in human QFT+ participants, making H107e a suitable candidate for subsequent product development and clinical testing. Interestingly, the response pattern was also in accordance with our published findings after immunization with 3xH107 in CB6F1 mice^18^, which is predominantly directed toward ESAT-6 and EspI and secondly toward MPT70 and MPT83. Although all the antigens in H107e induce protection on their own^18^, we were intrigued to see responses against both ESAT-6 and MTP70 as immunization with truncated versions of H107 has revealed that both the ESAT-6 repeats and the ‘MPT tail’ of MPT64, MPT70, and MPT83 are necessary for intact long-term protection^18^. This is in agreement with previous long-term data for ESAT-6-containing fusions^27^ and a recent study highlighting that MPT70 has a delayed in vivo expression profile and therefore might play a more prominent role during long-term infection^28^.

Multi-visit vaccination regimens are costly and inherently more complex to execute. This is particularly the case in many TB endemic areas, where logistics can be challenging and the risk of loss to follow-up is high, potentially compromising adequate vaccine coverage. Consequently, the Preferred Product Characteristic for novel TB vaccines set forth by WHO comprises a vaccination schedule with a minimal number of administrations^5^. Here we report on a highly efficacious single-visit regimen that, remarkably, has equivalent efficacy to a three-administration regimen. In a mouse model of tuberculosis, BCG+1xH107e was found to provide similar levels of protection as BCG+3xH107e against both short- and long-term Mtb infection. Additionally, the BCG+1xH107e single-dose co-administration regimen induced a long-lasting protective response even when used in conjunction with BCG revaccination, demonstrating that pre-existing immunity to distal BCG does not compromise this synergy. To our knowledge, this is the first demonstration of a protective single-visit TB vaccination regimen using subunit vaccines, which are otherwise administered in booster regimens with a minimal of two doses (and three in case of a BCG boosting approach). Related, a systematic review on the Human Papilloma Virus (HPV) 16/18 vaccine, administered in either a two (children aged 9–14) or three (older individuals) dose schedule, suggested that a single HPV vaccine dose was as effective as the multi-dose regimens in preventing HPV infection^6^. Although randomized controlled trials (RCT) are still scarce in this aspect, results from a recent RCT has shown non-inferiority in immunogenicity of a single dose HPV vaccine^29^. These efforts demonstrate that protective single-visit vaccination regimens, in this case against HPV, are pursued as a highly desirable and feasible strategy for broad vaccine implementation. Intriguingly, in our study, the single dose BCG+1xH107e regimen appeared to provide superior lung protection compared to the spleen at early infection stages (week 4), whereas the protection was significantly improved in both the spleen and lung at week 20. This finding is in agreement with the notion that BCG protects very well against bacterial dissemination by itself and indicates that the efficacy of the single-dose co-administration schedule probably relies on increased protection against local infection rather than protection against dissemination.

We found that BCG+1xH107e induced robust and long-lived H107e-specific CD4 T cells and that BCG co-administration appeared to limit the degree of T cell contraction from week 5 to 10 post immunization relative to H107e alone. However, BCG co-administration was overall found to have a minor effect in adjuvanting the H107-specific response compared to a three-dose regimen. This discrepancy can potentially be related to the availability of either vaccine in time and space. CAF01 (included in the H107e vaccine in these studies) is a depot forming adjuvant^30^, and although a single immunization with antigen can drive proliferation of adoptively transferred antigen-specific CD4 T cells up to 44 days post immunization (p.i.), the fraction of proliferating cells drops notably at 14 days relative to 3 days p.i.^31^. In line with this, biodistribution studies have shown that the majority of the antigen injected with CAF01 is cleared from the site of injection (SOI) by day 4 p.i. with only 2% of the initial dose remaining at the SOI by day 14. This is also reflected in the percentage of the antigen dose reaching the draining lymph node, where a notable drop can be observed within the first two weeks post immunization^31–33^. BCG is however known to exhibit slow replication and to persist long-term in both man^34^ and mice^35,36^. We found peak influx of neutrophils and inflammatory monocytes to occur around week 5 post BCG vaccination in mice in line with peak responses reported in literature^18,35^. At this time-point, the vast majority of the H107e antigen would be expected to be gone and thus unavailable for being co-adjuvanted. However, BCG has been shown to upregulate MINCLE/Clec4e (the ligand for TDB in CAF01) on myeloid cells^37^ and could thus render them susceptible for being targeted by H107e/CAF01 and lead to adjuvanted H017e-specific responses in a BCG+H107e co-administration schedule. Whether co-targeting to the same—or same type of—APCs by both components (BCG and H107e) is a requisite for the adjuvant effect warrants further investigation. Nevertheless, we speculate that BCG peak responses and H107e antigen availability should co-occur in order for a pronounced H107e adjuvanting effect to arise as a consequence of BCG co-administration. If so, at least two H107e/CAF01 administrations spaced 2–4 weeks apart would be required. Regardless of the modest adjuvanticity on T cell magnitude, single dose H107e+BCG still conferred protection equivalent to a BCG+3xH107e regimen.

In ref. ^18^, we reported that BCG and H107 co-administration led to reciprocal adjuvanticity and thus also observed an impact on the magnitude of the BCG-specific immune response. In the current study, we analyzed both innate and adaptive responses after BCG+1xH107e. Here, we found that the CAF01 component was the main driver in altering the innate cellular composition in the vaccine-draining lymph node and that different DC subsets, in particular CD8+ and CD11b+ DCs, constituted the main PCA loadings that contributed to the discrimination between the BCG vs. the BCG + CAF01 and BCG+1xH107e vaccine settings. DCs play a key role in CD4 T cell polarization, which are in part regulated through NOTCH signaling in T cells. Hence, differentiation along the Th1-Th17 axis is fine-tuned through upregulation of the NOTCH ligands Jagged1 and DLL4, respectively, on certain DC subsets—in particular CD11b+ and CD8a+ DCs along with lung CD103+ DCs^38–42^. It was therefore of interest to observe that the BCG+1xH107e single dose regimen led to the induction of BCG-specific Th17 cells that significantly expanded following Mtb challenge. Enhanced Th17 responses are also reported after mucosal delivery of BCG as well as attenuated Mtb vaccines^20,21,23,24^, and their induction might also rely on targeted delivery to CD11b+ and CD103 + DC subsets, which are key subsets within the lung^39,40^. Interestingly, the Th17 skewing observed in the current study would be in line with a ‘CAF01 imprint’^43,44^, but administration of CAF01 alone together with BCG did not skew the BCG-specific response towards Th17. Instead, this required the presence of both the adjuvant and antigen when co-administered with BCG (i.e., BCG+1xH107e). This is in line with our observation that the magnitude of the BCG-specific immune response increased with increasing doses of H107e in the BCG+3xH107e regimen. We, therefore, suggest that the Th17-skewing of BCG responses involves both a CAF01-dependent alteration of the innate cell composition in the draining lymph node as well as a bystander effect of “CAF01-imprinted” H107e-specific cells—potentially through increased local production of Th17 polarizing cytokines. Ultimately, this finding represents yet another example of Th17 cells being associated with improved protection against Mtb^20–25^ and warrants further investigation of the mechanisms behind the potential protective role of Th17 cells induced by co-administering BCG with H107e.

In order to confer population-level immunity and protection from infectious diseases, especially in Low-and Middle-income Countries, a single dose strategy would be highly preferable. Existing, efficacious single-dose vaccines include certain live vaccines such as the Yellow Fever vaccine^45^, Mumps, Measles and Rubella (MMR)^7,11^ and viral vectored Covid-19 vaccines^9,10^. While technically requiring two doses, the effectiveness of a single dose of the MMR vaccine has been estimated to be between 69 and 81% for prevention of clinical mumps and up to 95% for the prevention of clinical measles^7,11^. The murine data presented here suggests that an efficacious single visit TB vaccine regimen might be within reach, if the beneficial effects of BCG vaccination are combined with H107e. This would have important implications as it could substantially reduce the costs related to vaccine procurement and distribution, alleviate logistic constraints and secure vaccine access to the TB-endemic countries that need it most. The modifications introduced in H107e leading to significantly enhanced protein expression combined with a single dose approach could thus significantly reduce the costs associated with the completion of a TB vaccine regimen. Furthermore, our finding that BCG+1xH107e also protects in a BCG revaccination setting demonstrates proof of concept for the single visit regimen for both infant vaccination as well as adolescents and adults that are already BCG primed.

Collectively, our data exemplify how the cross-talk between BCG and subunit vaccines, including the adjuvant used, has profound consequences for the resulting immune responses and advocate for the feasibility of a single-dose TB vaccine regimen leveraging from the synergy between BCG and the complementing H107e subunit vaccine.

## Methods

### Antigen design and recombinant proteins

The H107 fusion protein is composed of eight Mtb antigens, of which ESAT-6 is repeated four times: PPE68-[ESAT-6]-EspI-[ESAT-6]-EspC-[ESAT-6]-EspA-[ESAT-6]-MPT64-MPT70-MPT83^18^ (Fig. 1a). H107e is identical to H107 except for deletion of amino acids 75-294 in EspI (Fig. 1a). Plasmids with the genes encoding H107 and H107e were transformed into *Eschericia coli* (*E. coli*) strain MAS78 gal^+^ thi rel^+^ Ara F´ (proAB ΔlacZlacY^-^lacI^q^1), and expression of H107 and H107e was induced with 1 mM isopropyl β-d-1-thiogalactopyranoside. The expression levels were evaluated by SDS-PAGE and western blot with monoclonal anti-MPT70 antibody at different time points after induction. All recombinant proteins were produced and purified in-house. Except for H107e produced in SSI’s *E. coli* production strain, all proteins contained a His-tag at the N-terminal end (MHHHHHH-) and the DNA constructs were codon-optimized for expression in *E. coli* and inserted into the pJ411 expression vector (ATUM, Menlo Park, CA, US) followed by transformation into *E. coli* BL21 (DE3) (Agilent, DK) competent cells. Proteins were then isolated from inclusion bodies in 3–12 L cultures and purified by metal chelate chromatography followed by anion-exchange chromatography. For the current study, TB10.4/Rv0288 and the fusion proteins H107 and H107e were produced. After purification, protein purity was estimated to be above 95% based on SDS-page followed by Coomassie staining and an anti-*E. coli* western blot. H107e had a recovery yield of 2.5–4.0 mg/L culture media, which was 2.5–5 fold higher than for H107 measured by the Bicinchoninic Acid Protein Assay. The potential impact of deleting the proline-rich fraction in the Espl/ Rv3876 antigen (Δ75-294) on antigen recognition in humans was determined by in silico MHC class II binding prediction of epitopes using the IEDB (https://www.iedb.org/). The amino acid sequence of Rv3876 was obtained from the Mycobrowser server (https://mycobrowser.epfl.ch) and analyzed for binding epitopes using the methods SMM-align^46^ and NN-align^47^. The prediction software identified 9-mer core peptides based on binding affinity (IC50 values given in nM) to the chosen HLA alleles. All epitopes identified by both methods and with an IC value < 500 nM were considered as binding epitopes. Prediction was performed in 27 common HLA DRA, DQ, and DP molecules as described by Greenbaum et al.^48^.

### Peptide pools

Peptide pools were obtained from JPT Peptide Technologies GmbH. Peptides were designed as 15-mers with 5-10 amino acids in overlap and a purity >80%. The proteins were covered by the following number of peptides: PPE68 (25 peptides), ESAT-6 (17 peptides), EspI (66 peptides), EspC (9 peptides), EspA (37 peptides), MPT64 (39 peptides), MPT70 (31 peptides), MPT83 (36 peptides).

### Human subjects and samples

Blood samples were obtained from the University of California San Diego, Antiviral Research Center Clinic. Ethical approval to carry out this work is maintained through the La Jolla Institute for Immunology Institutional Review Board. All participants provided written informed consent prior to participation in the study. We recruited 22 QFT + individuals and 10 TB negative controls (QFT-). QFT status was confirmed by an IFN-γ release assay (QuantiFERON Gold In-Tube, Cellestis). Further, subjects did not have any clinical or radiographic signs of active TB. Venous blood was collected in heparin-containing blood bags or tubes. PBMCs were purified from whole blood or 100 ml of leukapheresis samples by density-gradient centrifugation (Ficoll-Hypaque; Amersham Biosciences) according to the manufacturer’s instructions. PBMCs were cryopreserved in liquid nitrogen suspended in fetal bovine serum (Gemini Bio-Products) containing 10% (vol/vol) DMSO (Sigma-Aldrich).

### Fluorospot assay

T-cell responses to H107 and H107e were measured by IFN-γ Fluorospot assay (Mabtech), according to the manufacturer’s instructions. Briefly, Immobilon-FL PVDF 96-well plates (Mabtech) were coated overnight at 4 °C with mouse anti-human IFN-γ (clone 1-D1K). PBMCs were thawed and plated at a concentration of 200,000 cells per well and stimulated with the respective proteins and peptide pools at 37 °C in a humidified CO_2_ incubator for 22 h. As a positive control, 10 µg/ml phytohemagglutinin was used. In order to assess non-specific cytokine production, cells were also stimulated with culture media alone. All conditions were tested in triplicates. After incubation, cells were removed and plates were washed six times with 200 µl phosphate buffered saline (PBS) with 0.05% Tween 20 using an automated plate washer. After washing, anti-IFN-γ (7-B6-1-FS-BAM) diluted in PBS with 0.1% BSA was added to each well, and plates were incubated for 2 h at room temperature. The plates were washed again and then incubated with diluted fluorophores (anti-BAM-490) for 1 h at room temperature. After the final wash, plates were incubated with a fluorescence enhancer for 15 min. Spots were counted by computer-assisted image analysis (Mabtech IRIS, Mabtech). The responses were considered positive if they met all three criteria (I) the net spot forming cells per 10^6^ PBMC were >20, (ii) the stimulation index (i.e., fold above background) ≥2, and (iii) *p* ≤ 0.05 by two-sided Student’s *t* test^49^.

### Mice

Six-to-eight-week-old female CB6F1 (H2^b,d^) were obtained from Envigo (Netherlands). Mice were randomly assigned to cages upon arrival. Before the initiation of experiments, mice had at least 1 week of acclimatization in the animal facility. During the course of the experiment, mice had access to irradiated Teklad Global 16% Protein Rodent Diet (Envigo, 2916C) and water ad libitum. Mice were housed at an ambient temperature of 20–23 °C and 45–65% relative humidity on a 12 h/12 h light/dark cycle with 15 min dusk and dawn transition periods under Biosafety Level (BSL) II or III conditions in individually Type III ventilated cages (Scanbur, Denmark). Mice had access to nesting material (enviro-dri and soft paper wool; Brogaarden, Denmark) as well as enrichment (aspen bricks, paper house, corn, seeds, and nuts; Brogaarden, Denmark).

### Ethics for animal studies

Statens Serum Institut’s Animal Care and Use Committee approved all experimental procedures and protocols. All experiments were conducted in accordance with the regulations put forward by the Danish Ministry of Justice and Animal Protection Committees under license permit no. 2019-15-0201-00309 and in compliance with the European Union Directive 2010/63 EU.

### Immunization regimens

H107 and H107e were diluted in Tris-HCL buffer + 2% Glycerol (pH 7.2) and formulated in Cationic Adjuvant Formulation 1® (CAF®01) composed of 250 µg DDA/50 µg TDB per dose^19^. Final dose of H107 or H107e administered was 1 µg protein, unless otherwise indicated in the figure legends. BCG-Danish (1331) was diluted in PBS to an administered dose of 0.5 × 10^6^ BCG Colony Forming Units (CFU). For all immunizations, mice were subcutaneously (s.c.) injected with a volume of 200 µl, at the base of the tail. In the co-administration regimens, 0.5 × 10^6^ CFU BCG was administered s.c. on day −1, and the first immunization with adjuvanted protein was administered at the same site on day 0, for drainage to the same lymph node. This was the closest practically feasible model of a single visit strategy in the mouse model, given the physiological constrains. A pilot study, where the vaccines were administered on the same day, demonstrated that the vaccine material of the second vaccination was at risk of leaking out of the injection puncture site from the first vaccination. Spacing the two vaccinations by one day remedied this technical issue and is expected to have minimal effect on the resulting immune response given that the majority of antigen uptake after Ag/CAF01 immunization happens *after* day 1^28,29^ and that BCG persists for several weeks-months^32–34^. For all three dose regimens animals were immunized at 2-week intervals. Negative control mice were immunized with Tris-HCL buffer only or left non-vaccinated.

### Mycobacterial infections and enumeration of Mtb in organs

Ten weeks after the first immunization, mice were challenged with Mtb Erdman (ATCC 35801/TMC107). Mtb Erdman was cultured in Difco ™ Middlebrook 7H9 (BD) supplemented with 10% BBL ™ Middlebrook ADC Enrichment (BD) for two-three weeks using an orbital shaker (~110 rpm, 37 °C). Bacteria were harvested in log phase and stored at −80 °C until use. On the day of the experiment, the bacterial stock was thawed, sonicated for five minutes, thoroughly suspended with a 27G needle, and mixed with PBS to the desired inoculum dose. Using a Biaera exposure system controlled via AeroMP software, mice were challenged by the aerosol route with virulent Mtb Erdman in a dose equivalent to 50–100 CFUs.

In order to determine vaccine efficacy, Mtb CFUs were enumerated in lungs and spleens. Left lung lobes or spleens were homogenized in 3 mL MilliQ water containing PANTA™ Antibiotic Mixture (BD, cat.no. #245114) using GentleMACS M-tubes (Miltenyi Biotec). Tissue homogenates were serially diluted, plated onto 7H11 plates (BD), and grown for ~14 days at 37 °C and 5% CO_2_. CFU data were log-transformed before analyses.

### Preparation of single-cell suspensions

Spleens, lungs, and inguinal lymph nodes were aseptically harvested from euthanized mice and processed to extract single cell suspensions in accordance with established methodology^50^. Initially, lungs were homogenized in Gentle MACS tubes C (Miltenyi Biotec), followed by a 45 min collagenase digestion step (Sigma Aldrich; C5138) at 37 °C, 5% CO_2_. The lung homogenate, spleens, and lymph nodes were subsequently forced through 70-µm cell strainers (BD) with the plunger from a 3 mL syringe (BD). Cells were washed twice in cold RPMI or PBS followed by 5 min centrifugation at 700 × *g*. Finally, cells were resuspended in supplemented RPMI media containing 10% fetal calf serum (FCS). Cells were counted using an automatic Nucleocounter (Chemotec) and cell suspensions were adjusted to 2 × 10^5^ cells/well for ELISA and 1–2 × 10^6^ cells/well for flow cytometry.

### IFN-γ and IL-17A ELISA

Splenocytes were cultured in the presence of 2 µg/mL recombinant proteins or peptide pools (JPT) for 3 days. Supernatants were harvested and analyzed by a sandwich ELISA to determine the concentration of total IFN-γ or IL-17A. Microtiter plates (96-well; Maxisorb; Nunc) were coated with 1 µg/ml capture antibodies (IFN-γ: clone R4-6A2; BD Pharmingen. IL-17A: clone TC11-18H10.1; Biolegend) diluted in carbonate buffer. Free binding sites were blocked with 2% (w/v) skimmed milk powder (Natur Drogeriet, Matas, Denmark) in PBS. Culture supernatants were harvested after 72 h of incubation at 37°, 5% CO_2_, and microtiter plates were incubated overnight with samples diluted in PBS with 2% Bovine Serum Albumin (BSA, Sigma-Aldrich, MA, USA). IFN-γ was subsequently detected using a 0.1 µg/ml biotinylated rat anti-murine Ab (clone XMG1.2; BD Pharmingen) and recombinant IFN-γ (BD Pharmingen) as a standard, whereas IL-17A was detected using 0.25 µg/ml biotinylated anti-mouse IL-17A (BioLegend, clone: TC11-8H4) and recombinant IL-17A (BioLegend). Streptavidin HRP (BD Pharmingen, CA, US) diluted 1:5000 in PBS 1% BSA was used to detect bound biotinylated detection antibodies. The enzyme reaction was developed with 3,3′,5,5′-tetramethylbenzidine, hydrogen peroxide (TMB Plus; Kementec), stopped with 0.2 M H2SO4 solution and plates read at 450 nm with 620 nm background correction using an ELISA reader (Tecan Sunrise).

### Flow cytometry

Class II MHC Tetramers (I-A^d^:TB10.4_73-88_, I-A^b^:ESAT-6_4-17_) conjugated to PE or APC and corresponding negative controls (I-A^d^:hCLIP, I-A^b^:hCLIP) were provided by the NIH tetramer core facility (Atlanta, USA). Single-cell suspensions were stained with tetramers diluted 1:100 in FACS buffer (PBS + 1%FCS) containing 1:100 Fc-block (anti-CD16/CD32) for 30 min at 37 °C, 5% CO_2_. Tetramer staining was followed by surface staining, fixation, and eventually transcription factor staining as described below.

For intracellular cytokine analysis, splenocytes or lung cells were stimulated ex vivo with 2 µg/mL recombinant protein in the presence of 1 μg/ml anti-CD28 (clone 37.51) and anti-CD49d (clone 9C10-MFR4.B) for 1 h at 37 °C, 5% CO_2_ followed by the addition of Brefeldin A to 10 µg/mL (Sigma Aldrich; B7651-5mg) and 5-6 h of additional incubation at 37 °C, after which the cells were kept at 4 °C until staining the subsequent day.

Cells were then stained for surface markers diluted in 50% brilliant stain buffer (BD, 566349) and fixable viability dye eFlour^TM^506 (1:500) or eFlour^TM^780 (1:500) (both eBioscience) at 4 °C for 20 min. Cells were subsequently fixed and permeabilized with the Cytofix/Cytoperm Solution Kit (BD Biosciences) as per manufacturer’s instructions followed by ICS for IFN-γ, TNF, IL-2, and/or IL-17A at 4 °C for 30 min. Subsequent or independent transcription factor staining followed by additional fixation and permeabilization using the Foxp3/Transcription Factor Staining Buffer Set (eBioscience™; 00-5523-00) as per manufacturer’s instructions followed by RORγT staining at 4 °C for 30 min. Fluorescence minus one control was performed to set boundary gates for selected markers. Cells were acquired on a BD LSRFortessa and the FSC files were manually gated with FlowJo v10 (Tree Star). The following antibodies were used for the flowcytometric phenotyping of innate populations: CD11b-PE (BD Biosciences, clone: M1/70, catalog #553311, 1:200), CD11c-BV421 (BD Biosciences, clone: HL3, catalog #562782, 1:200), CD8a (BD Biosciences, clone: 53-6.7, catalog #563332, 1:200), CD103-PE-Dazzle594 (Biolegend, clone: 2E7, catalog # 121430, 1:100), Ly-6C-APC-Cy7 (Biolegend, clone: HK1.4, catalog #128025, 1:200), Ly-6G-PerCp-Cy5.5 (Biolegend, clone: 1A8, catalog #127616, 1:200), F4/80-APC (eBiosciences, clone: BM8, catalog #13-4801-82, 1:200), MCH-II-AF488 (Biolegend, clone: M5/114.15.2, catalog #107615, 1:300), CD3-BV605 (Biolegend, clone: 17A2, catalog #100237, 1:100), CD19-BV711 (Biolegend, clone: 6D5, catalog #115555, 1:400). For characterization of T cell responses, the following antibodies were used: CD3-BV650 (Biolegend, clone: 17 A2, catalog #100229, 1:100), CD3-BV605 (BD Biosciences, clone: 145-2C11, catalog #563004, 1:100), CD4-BV510 (Biolegend, clone: RM4.5, catalog #100559, 1:500), CD4-BV786 (BD Biosciences, clone GK1.5, catalog #563331, 1:400), CD8-BV650 (BD Biosciences, clone: 53-6.7, catalog #563234, 1:400), CD19-BV510 (Biolegend, clone: 6D5, catalog #115545, 1:150), CD19-PerCP-Cy5.5 (BD Biosciences, clone: 1D3, catalog #551001, 1:500), CD44-Alx700 (Biolegend, clone: IM7, catalog #103026, 1:150), KLRG1-BV711 (BD Biosciences, clone: 2F1, catalog #564014, 1:100), CXCR3-BV421 (Biolegend, clone: CXCR3-173, catalog #126529, 1:100), CXCR3-PerCP/Cy5.5 (ThermoFisher, clone: CXCR3-173, catalog #45-1831-82, 1:100), IFN-γ-PE-Cy7 (eBioscience, clone: XMG1.2, catalog #25-7311-82, 1:200), IFN-γ-BV421 (BD Biosciences, clone: XMG1.2, catalog #563376, 1:200), TNF-PE (eBioscience, clone: MP6-XT22, catalog #12-7321-82, 1:200), IL-2-APC/Cy7 (BD Biosciences, clone: JES6-5H4, catalog #560547, 1:100), IL-17A-BV421 (Biolegend, clone: TC11-18H10.1, catalog # 506926, 1:200), RORγT-PE/CF594 (BD Biosciences, clone: Q31-378, catalog #562684, 1:20).

When identifying antigen-specific cell percentages, the medium-only stimulation condition was subtracted from antigen stimulation for individual samples. For Boolean gating analyses, this media background was subtracted from each Boolean gate individually.

### Statistical analyses

All graphical visualizations and statistical tests were done using GraphPad Prism v8 or v9. PCA analyses were made in RStudio (v2022.07.2.0). Unless otherwise stated, One-Way Analysis of Variance (ANOVA) using Dunnett’s multiple comparison test (comparing to control mice only) or Tukey’s Multiple Comparison test (comparing across all groups) was used to evaluate significant differences between more than two vaccine groups. CFU values were log-transformed before statistical analysis. The specific statistical test used is stated in the figure legends. A *p*-value above 0.05 was considered not significantly different.

### Reporting summary

Further information on research design is available in the Nature Research Reporting Summary linked to this article.

## Supplementary information

Supplementary Information

Reporting Summary

## Data Availability

The data that support the findings of this study are available from the corresponding author upon request. There are no restrictions on data availability.
