# Supplementary material for: A protective, single-visit TB vaccination regimen by co-administration of a subunit vaccine with BCG
Source: NPJ Vaccines. 2023 May 9;8:66. doi: 10.1038/s41541-023-00666-2 (PMC10169149; doi:10.1038/s41541-023-00666-2)
Supplement: Supplementary file 1 — Supplementary Information [file 41541_2023_666_MOESM1_ESM.pdf]

Supplementary Figure 1.

a

HLA Allele	5-13	61-68	241-249	243-251	253-261	277-285	314-322	384-392	385-293	401-409	417-425	433-441	441-449	444-452	449-457	468-476	485-493	488-496	491-499	494-502	505-513	507-515	519-527	521-529	533-541	542-550	546-552	549-557	553-561	556-562	559-567	562-570	564-572	596-602	626-634	630-638	641-649	644-652	649-657	650-658
HLA-DPA1*01/DPB1*04:01																																								
HLA-DPA1*01:03/DPB1*02:01													•	•										•																
HLA-DPA1*02:01/DPB1*01:01																																				•		•		
HLA-DPA1*02:01/DPB1*05:01																																						•		
HLA-DPA1*02:01/DPB1*14:01																																								
HLA-DPA1*03:01/DPB1*04:02														•						•																				
HLA-DQA1*01:01/DQB1*05:01																																								
HLA-DQA1*01:02/DQB1*06:02							•		•			•		•		•	•	•	•	•									•		•		•			•				
HLA-DQA1*03:01/DQB1*03:02																																								
HLA-DQA1*04:01/DQB1*04:02																•																								•
HLA-DQA1*05:01/DQB1*02:01																																							•	
HLA-DQA1*05:01/DQB1*03:01		•	•	•	•	•	•				•				•	•		•			•	•		•	•		•	•	•		•		•	•	•	•			•	•
HLA-DRB1*01:01	•	•	•	•	•		•	•		•	•	•	•	•	•		•	•	•	•	•	•	•	•		•	•			•		•	•	•	•	•	•		•	•
HLA-DRB1*03:01																																								
HLA-DRB1*04:01		•			•	•	•	•				•	•	•	•		•	•				•	•	•															•	
HLA-DRB1*04:05		•						•				•												•			•													•
HLA-DRB1*07:01								•				•	•				•		•				•	•				•		•					•		•		•	•
HLA-DRB1*08:02									•																				•	•										
HLA-DRB1*09:01	•	•	•	•	•			•		•		•					•	•			•	•	•	•		•		•		•		•	•		•	•	•			•
HLA-DRB1*11:01	•							•	•	•												•				•											•			
HLA-DRB1*12:01																							•	•															•	
HLA-DRB1*13:02													•	•				•						•	•			•		•				•						
HLA-DRB1*15:01						•																					•	•		•							•		•	
HLA-DRB3*01:01										•				•								•		•	•											•				
HLA-DRB3*02:02																																								
HLA-DRB4*01:01									•				•	•						•										•	•	•						•		
HLA-DRB5*01:01		•						•			•	•	•											•																
**Total**	3	6	3	3	4	3	4	7	4	4	3	7	7	8	3	3	5	6	3	4	3	6	5	11	3	3	4	5	3	7	3	3	4	3	4	6	5	3	7	6

Columns: Rv3876 Amino Acids

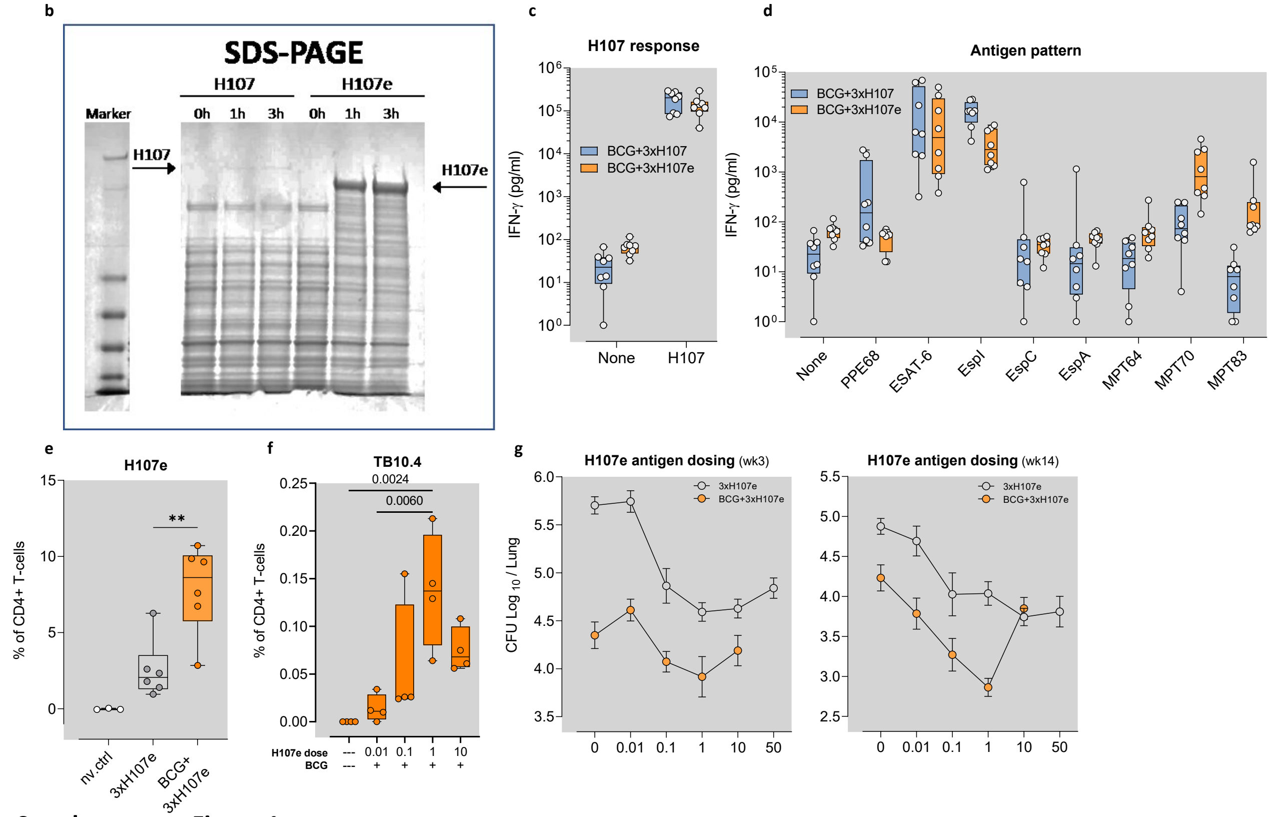

Supplementary Figure 1

a In silico MHC class II prediction of binding epitopes in Rv3876. The amino acid sequence of Rv3876 was obtained from the Mycobrowser server and analyzed for potential HLA binding epitopes using the SMM-align and NN-align methods. Columns in the checkerboard table show 9-mer core peptides identified as binding epitopes within antigenic stretches of the Rv3876 antigen. Each row shows identified HLA binding alleles among 27 common HLA DRA, DQ and DP molecules. IC50 values <500nM were employed as cutoff for identified binding epitopes (shown as dots). Area marked by orange box signify the deleted proline-rich fraction in the Rv3876 antigen (Δ75-294) in H107e relative to H107. **b** Full size SDS-page gel showing the expression of the H107 and H107e constructs (directly from OD adjusted cultures) at 0, 1- and 3-hours post-induction. Protein purity (after purification) was estimated to be above 95% based on SDS-page followed by Coomassie staining and an anti-E. coli western blot. H107e had a recovery yield of 2.5 – 4.0 mg/L culture media, which was 2.5 – 5-fold higher than for H107. **C** H107/H107e specific IFN-γ production by splenocytes from animals vaccinated with BCG+3xH107/CAF®01 or BCG+3xH107e/CAF®01, as measured by ELISA. Data graphed as box plots with line at median, boxes indicating interquartile range, and whiskers delineating minimum and maximum values (n=8). **d** IFN-γ release after ex vivo stimulation with single antigen components of H107(e) by splenocytes from animals vaccinated with BCG+3xH107/CAF®01 or BCG+3xH107e/CAF®01, as measured by ELISA. Data graphed as box plots with line at median, boxes indicating interquartile range, and whiskers delineating minimum and maximum values (n=8). **e** Cumulative frequency of cytokine-positive (IFN-γ, TNF-α, IL-2 and/or IL-17A) CD4 T cells in spleen after stimulation with H107e protein in mice immunized with 3x H107e/CAF®01 compared to BCG+3x H107e/CAF®01. Box plots with line at median, boxes indicating interquartile range, and whiskers showing minimum and maximum values (n=3-6). Statistical significance of group differences determined by Student's T-test. **f** Cumulative frequency of cytokine-positive CD4 T cells in spleen after stimulation with TB10.4 protein in mice immunized with different H107e doses administered 3xH107e/CAF®01 compared to BCG+3x H107e/CAF®01. Box plots with line at median, boxes indicating interquartile range, and whiskers showing minimum and maximum values (n=4). One-way ANOVA with Tukey's post comparison test. **g** Lung bacterial burden at week three (left) and fourteen (right) post aerosol challenge with Mtb Erdman in mice immunized with 3xH107e/CAF®01 in a range of doses administered with or without BCG co-administration. Data plotted as mean ± SEM (n=6-8).

Supplementary Figure 2

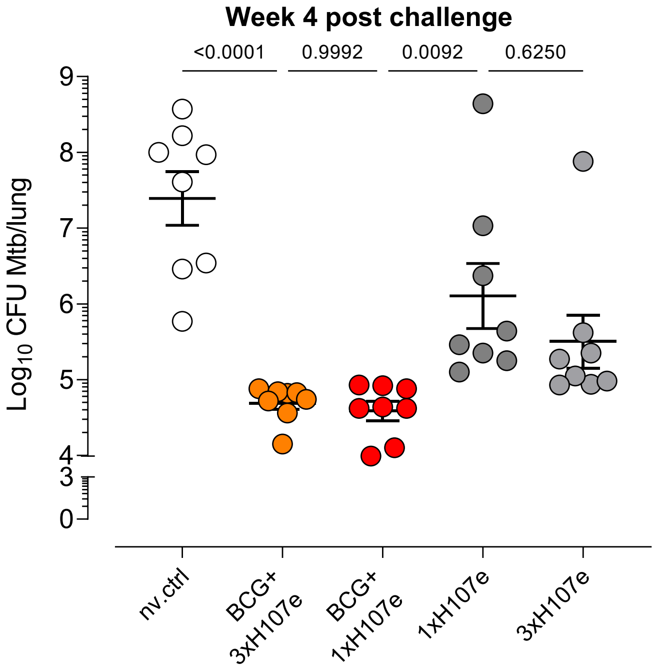

Supplementary Figure 2

Independent repeat study showing that co-administration schedules with single and triple administrations (BCG+1xH107e vs BCG+3xH107e) leads to similar levels of protection. Lung bacterial loads 4 weeks after Mtb challenge. Line and error bars indicate mean and SEM, respectively (n=8). Statistical significance of group differences determined via One-way ANOVA and Tukey's multiple comparison test.

Supplementary Figure 3

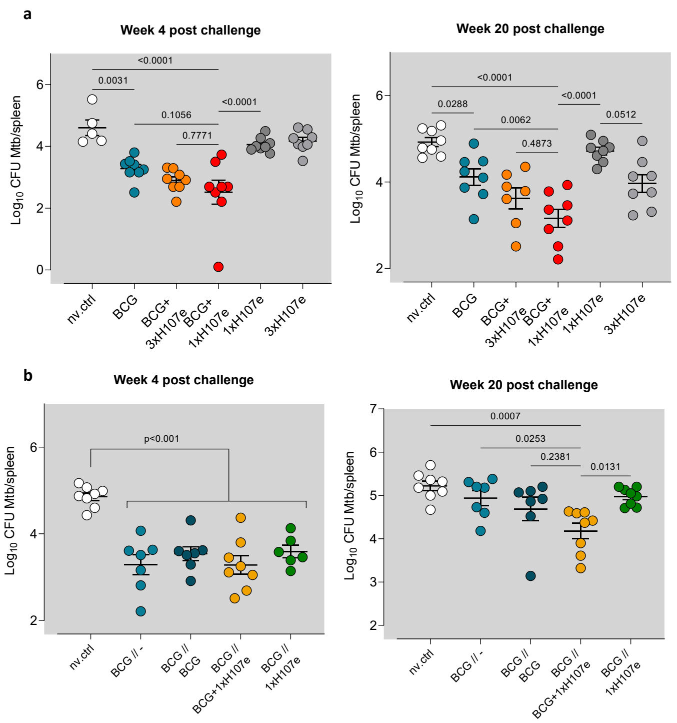

Supplementary Figure 3

a Spleen bacterial loads of the animals from the study described in **Figure 2**, 4 (left panel) and 20 weeks (right panel) after challenge with Mtb. **b** Spleen bacterial loads of the animals from the study described in **Figure 3**, 4 (left panel) and 20 weeks (right panel) after challenge with Mtb. Line and error bars depict mean and SEM. Statistical significance of group differences was determined via one-way ANOVA and Tukey's multiple comparison test

Supplementary Figure 4

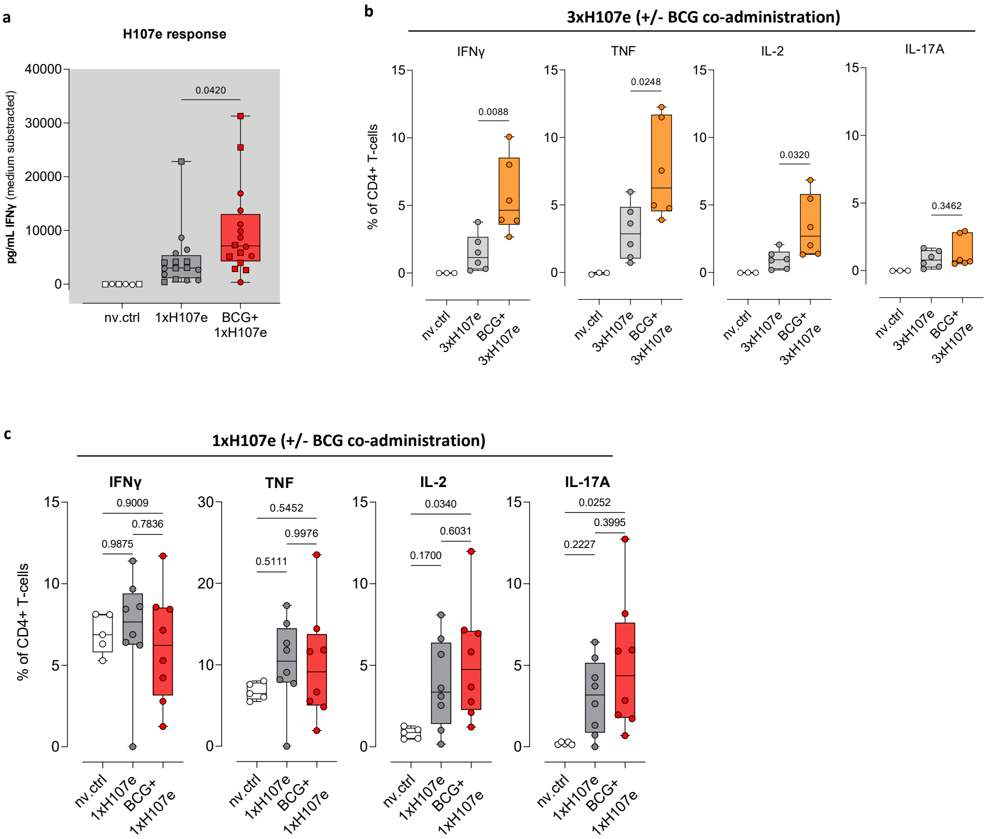

Supplementary Figure 4

a H107e specific IFN-γ production by splenocytes from animals vaccinated with 1xH107e/CAF®01 or BCG+1xH107e/CAF®01, as measured by ELISA. Data pooled from two independent studies, differentiated by symbol shape, and graphed as box plots with line at median, boxes indicating interquartile range, and whiskers delineating minimum and maximum values. **b** Comparison of individual cytokine responses between the animals vaccinated with 3xH107e/CAF®01 versus BCG+3xH107e/CAF®01, 1 week after vaccination. Data graphed as box plots with line at median, boxes indicating interquartile range, and whiskers delineating minimum and maximum values. Statistical significance of group differences determined via unpaired t-test. **c** Comparison of individual H107e-specific cytokine responses between the animals vaccinated with 1xH107e versus BCG+1xH107e, 4 weeks after Mtb aerosol infection. Data graphed as box plots with line at median, boxes indicating interquartile range, and whiskers delineating minimum and maximum values. Statistical significance of group differences determined via One-way ANOVA with Tukey's multiple comparisons test. n=8, though n=5 in nv. ctrl (non-vaccinated control).

Supplementary Figure 5

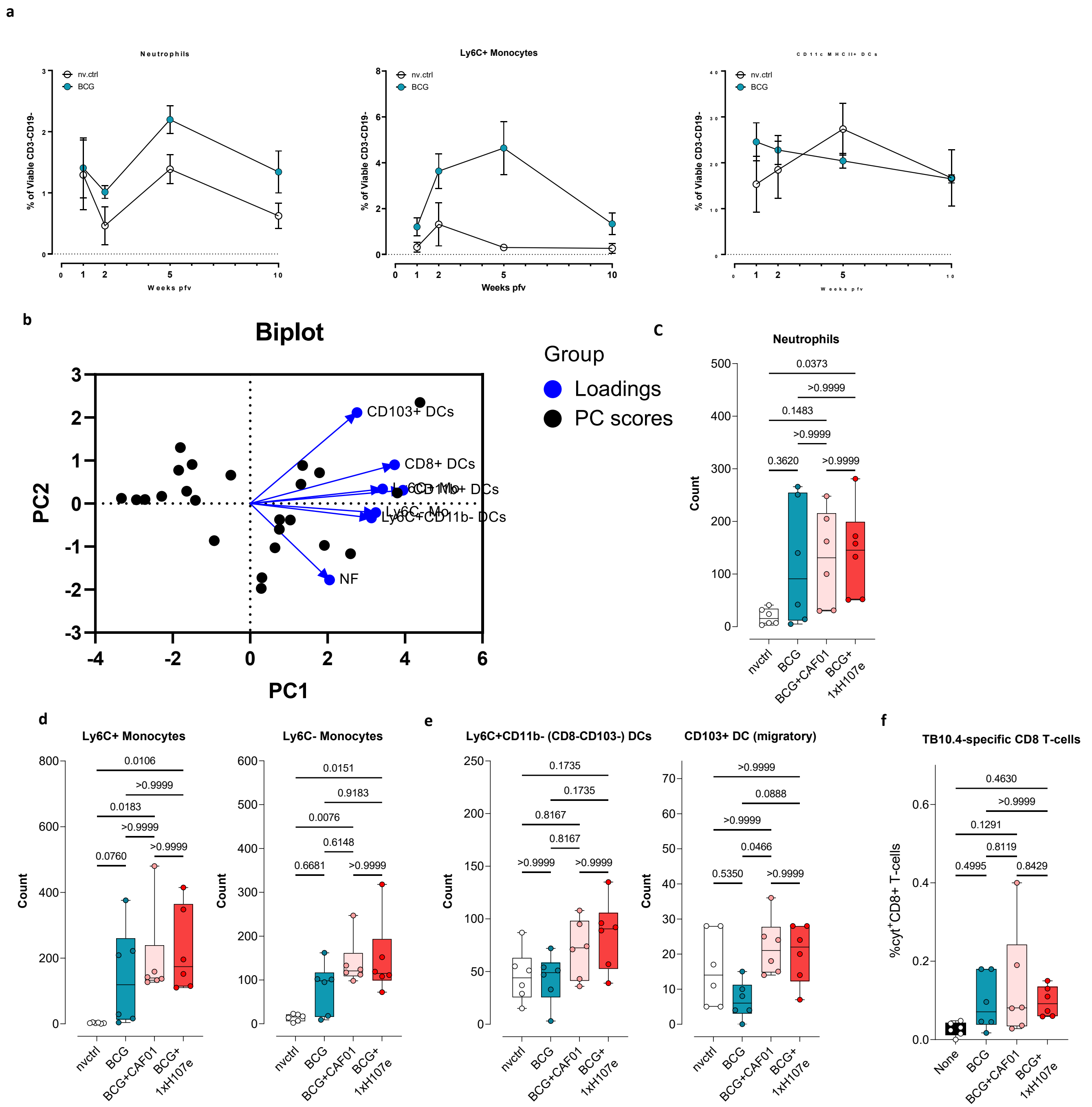

Supplementary Figure 5

a Frequencies of neutrophils (left), Ly-6C+ monocytes (middle) and MHC II+ DCs (right) in inguinal lymph nodes draining the vaccination site at week 1, 2, 5 and 10 post BCG vaccination and in non-vaccinated control mice. **b** Loadings from PCA on neutrophil, Ly6C- and Ly6C+ monocyte, CD8a+-resident, CD103+-migratory, CD11b+ and Ly6C+ DC cell numbers in vaccine-draining lymph nodes at week 5 post immunization among BCG, BCG+CAF®01 and BCG+1x H107e/CAF®01 immunized relative to non-immunized mice. **c** Number of neutrophilic cells **d** Number of Ly-6C+ (left) and Ly-6C- (right) monocytes. **e** Number of DC subsets. Ly-6C+CD11b- (CD8a-CD103-) DCs (left) and CD103+ migratory DCs (right). **f** frequency of TB10.4-specific cytokine producing CD8 T cells. Data are in all cases graphed as box plots showing median ± IQR with whiskers signifying the range of responses. Statistical significant differences between groups determined using Kruskall-Wallis and Dunn’s multiple comparison test, n=6.

Gating Strategy for innate cell populations – Figure 5 & Supplementary Figure 5

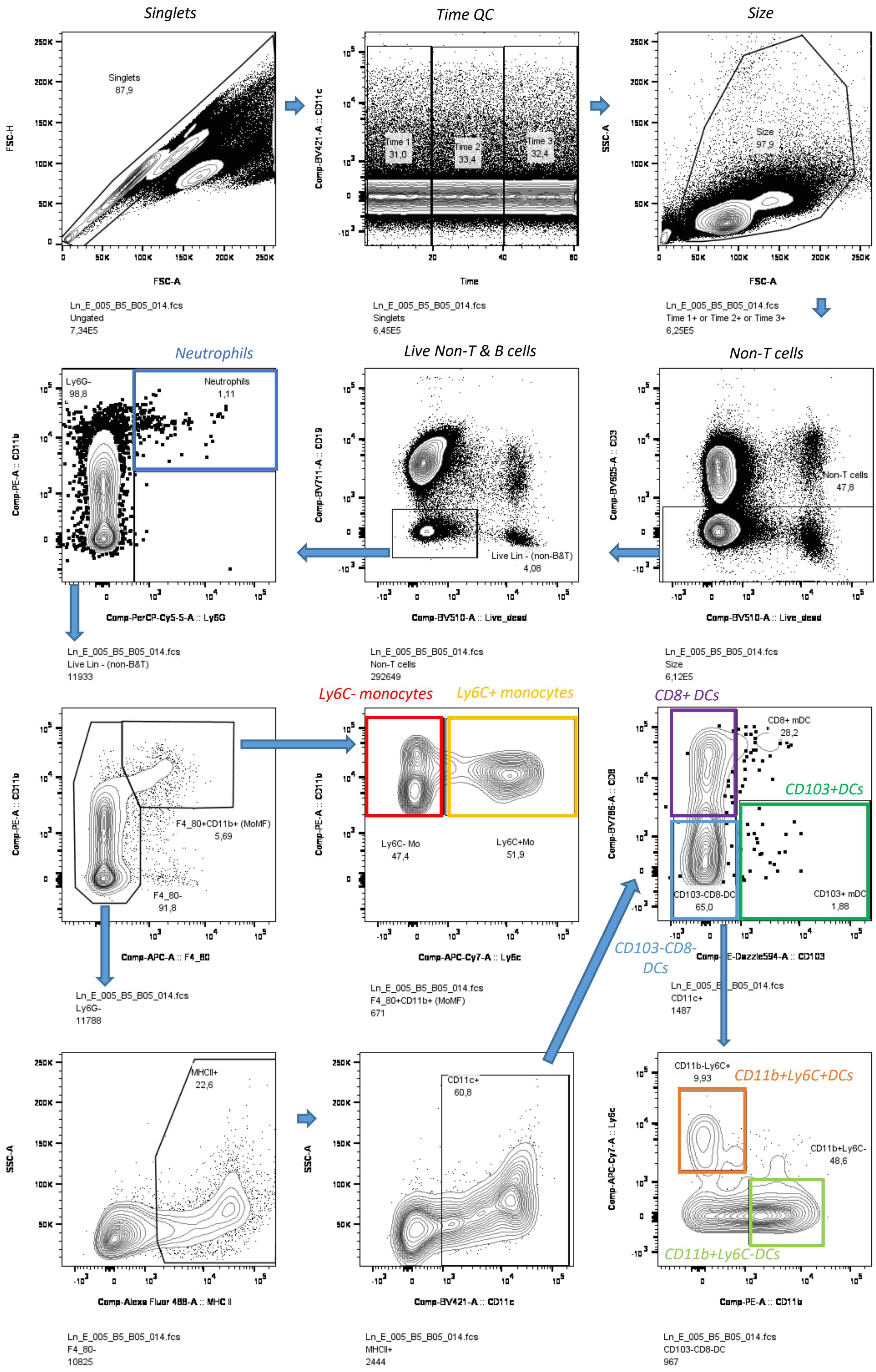

Gating Strategy for Cytokine +ve CD4 T cells

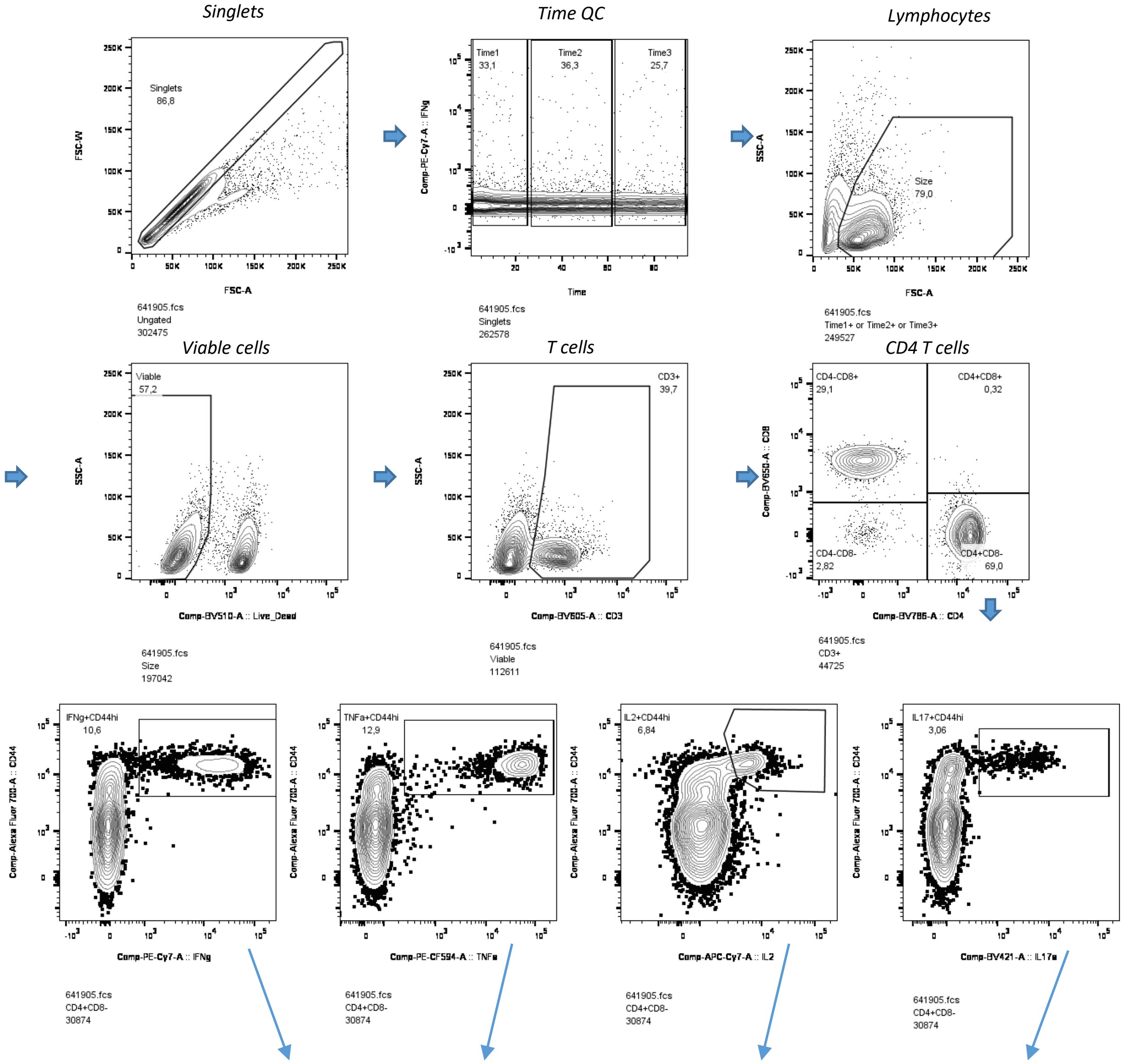

- Cytokine +ve: IFN-γ *or* TNF *or* IL-2 *or* IL-17A
- Cytokine co-expression profiles: Combination gates
