# Supplementary material for: A protective, single-visit TB vaccination regimen by co-administration of a subunit vaccine with BCG
Source: NPJ Vaccines. 2023 May 9;8:66. doi: 10.1038/s41541-023-00666-2 (PMC10169149; doi:10.1038/s41541-023-00666-2)
Supplement: Supplementary file 2 — Reporting Summary [file 41541_2023_666_MOESM2_ESM.pdf]

nature portfolio

Corresponding author(s): Rasmus Mortensen

Last updated by author(s): Mar 29, 2023

Reporting Summary

Nature Portfolio wishes to improve the reproducibility of the work that we publish. This form provides structure for consistency and transparency in reporting. For further information on Nature Portfolio policies, see our Editorial Policies and the Editorial Policy Checklist.

Statistics

For all statistical analyses, confirm that the following items are present in the figure legend, table legend, main text, or Methods section.

n/a	Confirmed	
☐	☒	The exact sample size (*n*) for each experimental group/condition, given as a discrete number and unit of measurement
☐	☒	A statement on whether measurements were taken from distinct samples or whether the same sample was measured repeatedly
☐	☒	The statistical test(s) used AND whether they are one- or two-sided<br>*Only common tests should be described solely by name; describe more complex techniques in the Methods section.*
☒	☐	A description of all covariates tested
☐	☒	A description of any assumptions or corrections, such as tests of normality and adjustment for multiple comparisons
☐	☒	A full description of the statistical parameters including central tendency (e.g. means) or other basic estimates (e.g. regression coefficient) AND variation (e.g. standard deviation) or associated estimates of uncertainty (e.g. confidence intervals)
☐	☒	For null hypothesis testing, the test statistic (e.g. *F*, *t*, *r*) with confidence intervals, effect sizes, degrees of freedom and *P* value noted<br>*Give P values as exact values whenever suitable.*
☒	☐	For Bayesian analysis, information on the choice of priors and Markov chain Monte Carlo settings
☒	☐	For hierarchical and complex designs, identification of the appropriate level for tests and full reporting of outcomes
☒	☐	Estimates of effect sizes (e.g. Cohen's *d*, Pearson's *r*), indicating how they were calculated

Our web collection on statistics for biologists contains articles on many of the points above.

Software and code

Policy information about availability of computer code

Data collection	N/A
Data analysis	N/A

For manuscripts utilizing custom algorithms or software that are central to the research but not yet described in published literature, software must be made available to editors and reviewers. We strongly encourage code deposition in a community repository (e.g. GitHub). See the Nature Portfolio guidelines for submitting code & software for further information.

Data

Policy information about availability of data

All manuscripts must include a data availability statement. This statement should provide the following information, where applicable:

- Accession codes, unique identifiers, or web links for publicly available datasets
- A description of any restrictions on data availability
- For clinical datasets or third party data, please ensure that the statement adheres to our policy

The raw data that support the findings of this study are available from the corresponding author upon request. There are no restrictions on data availability.

Field-specific reporting

Please select the one below that is the best fit for your research. If you are not sure, read the appropriate sections before making your selection.

☒ Life sciences ☐ Behavioural & social sciences ☐ Ecological, evolutionary & environmental sciences

For a reference copy of the document with all sections, see nature.com/documents/nr-reporting-summary-flat.pdf

Life sciences study design

All studies must disclose on these points even when the disclosure is negative.

Sample size	In our standardized murine aerosol Mtb challenge model, we powered the experiments for detecting a treatment effect (protection) of 0.5 log10 CFU reduction in lungs compared to non-treated controls with a type I error rate of 5% (α=0.05), a power of 80% and standard deviation of 0.35 log10 CFU (based on previous experiments). This results in n=8 mice per group for primary analysis.<br>For comparative T cell magnitude and phenotypical analysis, 4-8 mice per group was used.
Data exclusions	No data was excluded from the analysis
Replication	Results are presented from individual experiments or, if indicated, combined into a single data representation with symbols to indicate data points from independent experimental replicates.<br>In figure legends it is stated, if representative data is shown.<br>Main conclusion is supported by a minimun of two independent experiments
Randomization	Mice were randomly assigned to cages upon arrival to our animal facility. For each time point in the study, mice were randomly selected for primary analysis.
Blinding	The investigator was not involved in CFU data collection. Organ homogenization, plating, and CFU counting was performed by an experienced technician, who was not involved in study design and/or data analysis. The investigator was, however, not blinded during data analysis and interpretation.

Reporting for specific materials, systems and methods

We require information from authors about some types of materials, experimental systems and methods used in many studies. Here, indicate whether each material, system or method listed is relevant to your study. If you are not sure if a list item applies to your research, read the appropriate section before selecting a response.

Materials & experimental systems

n/a	Involved in the study	
☐	☒	Antibodies
☒	☐	Eukaryotic cell lines
☒	☐	Palaeontology and archaeology
☐	☒	Animals and other organisms
☐	☒	Human research participants
☒	☐	Clinical data
☒	☐	Dual use research of concern

Methods

n/a	Involved in the study	
☒	☐	ChIP-seq
☐	☒	Flow cytometry
☒	☐	MRI-based neuroimaging

Antibodies

Antibodies used

The following antibodies were used for the flowcytometric phenotyping of innate populations: CD11b-PE (BD Biosciences, clone: M1/70, catalog #553311, 1:200), CD11c-BV421 (BD Biosciences, clone: HL3, catalog #562782, 1:200), CD8a (BD Biosciences, clone: 53-6.7, catalog #563332, 1:200), CD103-PE-Dazzle594 (Biolegend, clone: 2E7, catalog # 121430, 1:100), Ly-6C-APC-Cy7 (Biolegend, clone: HK1.4, catalog #128025, 1:200), Ly-6G-PerCp-Cy5.5 (Biolegend, clone: 1A8, catalog #127616, 1:200), F4/80-APC (eBiosciences, clone: BM8, catalog #13-4801-82, 1:200), MCH-II-AF488 (Biolegend, clone: M5/114.15.2, catalog #107615, 1:300), CD3-BV605 (Biolegend, clone: 17A2, catalog #100237, 1:100), CD19-BV711 (Biolegend, clone: 6D5, catalog #115555, 1:400).

For characterization of T cell responses, the following antibodies were used: CD3-BV650 (Biolegend, clone: 17A2, catalog #100229, 1:100), CD3-BV605 (BD Biosciences, clone: 145-2C11, catalog #563004, 1:100), CD4-BV510 (Biolegend, clone: RM4.5, catalog #100559, 1:500) , CD4-BV786 (BD Biosciences, clone GK1.5, catalog #563331, 1:400), CD8-BV650 (BD Biosciences, clone: 53-6.7, catalog #563234, 1:400), CD19-BV510 (Biolegend, clone: 6D5, catalog #115545, 1:150), CD19-PerCP-Cy5.5 (BD Bbiosciences, clone: 1D3, catalog #551001, 1:500), CD44-Alx700 (Biolegend, clone: IM7, catalog #103026, 1:150), KLRG1-BV711 (BD biosciencesBiosciences, clone: 2F1, catalog #564014, 1:100), CCXCR3-BV421 (Biolegend, clone: CXCR3-173, catalog #126529, 1:100), CXCR3-PerCP/Cy5.5 (ThermoFisher, clone: CXCR3-173, catalog #45-1831-82, 1:100), IFN-γ-PE-Cy7 (eBioscience, clone: XMG1.2, catalog #25-7311-82, 1:200), IFN-γ-BV421 (BD Biosciences, clone: XMG1.2, catalog #563376, 1:200), TNF-PE (eBioscience, clone: MP6-XT22, catalog #12-7321-82, 1:200), IL-2-APC/Cy7 (BD bBiosciences, clone: JES6-5H4, catalog #560547, 1:100), IL-17A-BV421

	(Biolegend, clone: TC11-18H10.1, catalog # 506926, 1:200), , RORγT-PE/CF594 (BD Biosciences, clone: Q31-378, catalog #562684, 1:20).
Validation	Validation in the target species was performed by the supplier of the antibody.

Animals and other organisms

Policy information about studies involving animals; ARRIVE guidelines recommended for reporting animal research

Laboratory animals	CB6F1 mice (female BALB/c x male C57BL/6 F1 offspring, 'Envigo' Laboratories, The Netherlands). Mice were females, 6-10 weeks old when the experiments were initiated and kept in cages of 8 animals or less. Animals had access to chow and drinking water ad libitum during the experiments.
Wild animals	N/A
Field-collected samples	N/A
Ethics oversight	Experiments were conducted in accordance with the regulations set forward by the Danish Ministry of Justice and animal protection committees by Danish Animal Experiments Inspectorate Permit 2019-15-0201-00309 in compliance with European Community Directive 2010/63/EU of the European parliament and of the council of 22 September 2010 on the protection of animals used for scientific purposes. The experiments were approved by the local animal protection committee at Statens Serum Institut, IACUC, headed by DVM Kristin Engelhart Illigen.

Note that full information on the approval of the study protocol must also be provided in the manuscript.

Human research participants

Policy information about studies involving human research participants

Population characteristics	We enrolled 22 QFT+ (QuantiFERON Gold In-Tube, Cellestis) and 10 QFT- individuals at the Antiviral Research Center Clinic, University of California San Diego (UCSD). Subjects did not have any clinical or radiographic signs of active TB. None of the study subjects endorsed vaccination with BCG, or had laboratory evidence of HIV or Hepatitis B. QFT+ subjects were between 18-61 years old (median 39) and 55% were male, and QFT- were between 34-60 (median 46) with 80% male.
Recruitment	Participants were recruited on the basis of their history of a tuberculin skin test (TST) through flyers at the Antiviral Research Center Clinic, UCSD. Mtb infection was determined by QuantiFERON. A physical exam and/or chest X-ray ruled out active tuberculosis.
Ethics oversight	All participants provided written informed consent to the study and ethical approval to carry out the work is maintained through the La Jolla Institute for Immunology Institutional Review Board.

Note that full information on the approval of the study protocol must also be provided in the manuscript.

Flow Cytometry

Plots

Confirm that:

☒ The axis labels state the marker and fluorochrome used (e.g. CD4-FITC).

☒ The axis scales are clearly visible. Include numbers along axes only for bottom left plot of group (a 'group' is an analysis of identical markers).

☒ All plots are contour plots with outliers or pseudocolor plots.

☒ A numerical value for number of cells or percentage (with statistics) is provided.

Methodology

Sample preparation	Single cell suspensions from tissue samples were prepared by mechanically forcing the tissue through a 100 um cell strainer. For lung samples, a pre-processing step was necessary in which lungs were ground using Auto-MACS C-tubes (Miltenyi Biotec) and treated with collagenase IV for 30-60 minutes at 37 degrees, 5% CO_2. Cells were washed twice in RPMI and then resuspended in RPMI + 10% FCS. Cells were at this point on single cell level and ready for staining analysis.
Instrument	Samples were analyzed with a BD LSRFortessa™ using a BD™ High Throughput Sampler (HTS).
Software	Data management was carried out with BD FACSDiva™ v6.2 software. Data was analyzed using FlowJo software v.10 (Tree Star, Ashland, OR, USA).
Cell population abundance	No cell sorting was performed with FACS.
Gating strategy	For T-cell analyses, cells were gated for singlets (FSC-A/FSC-H), Time (QC, time/SSC-A), lymphocytes (FSC-A/SSC-A), live cells (Viability dye [APC-eF780 or BV510]/SSC-A), T cells (CD3[BV605 or BV650]/SSC-A) and a CD4/CD8 T cell gate (CD4+ [BV786 or BV510] , CD8+ [BV650]). Subsequently, CD44hi [AF-700] antigen specific cells were either gated by tetramer staining (MCH II

tetramer [PE or BV421]) specific T cells, or cytokine producing T cells (IFNg [PE/Cy7], IL-2 [APC/Cy7], TNF [PE/APC], IL-17 [BV421]) or (IFNg [PE/Cy7], IL-2 [APC/Cy7], TNF [PE-Dazzle594], IL-17 [BV421]. An 'any' cytokine producing cell gate was created via Boolean 'OR' gating in FlowJo. Gatings for combinatorial cytokine expression analysis were created using the 'create combination gates' option in FlowJo.
Antigen-specific T cells were further characterized for their expression of T-bet [e660] and/or RORgT [PE-CF594].

For innate cell population phenotyping, cells were gated for singlets (FSC-A/FSC-H), Time (QC, time/BV421), size (FSC-A/SSC-A), non-T cells (Viability dye [BV510]/CD3 [BV605]), Live non B & T cells (Viability dye [BV510]/CD19 [BV711]), Ly-6G+ (neutrophils) vs Ly-6G- (Ly-6G [PerCp-Cy5.5]/CD11b [PE]), F4/80+CD11b+ (MoMF) vs F4/80- (F4/80 [APC]/CD11b [PE]), Ly-6C- vs Ly-6C+ Mo (Ly-6C [APC-Cy7)/CD11b [PE]), F4/80-MHC II+ (MHC II [AF488]/SSC-A), CD11c+ DCs (CD11c [BV421]/SSC-A), CD8+ DCs vs CD103+ DCs vs CD8-CD103- DCs (CD103 [PE-Dazzle594]/CD8a [BV786], CD11b-Ly6C+ vs CD11b+Ly6C- (CD11b [PE]/Ly6C[APC-Cy7]).

Fixable Viability dye (viability-eF780 and eF506) was used to discriminate dead cells for all tetramer stainings and for ICS when possible.

☒ Tick this box to confirm that a figure exemplifying the gating strategy is provided in the Supplementary Information.
